# Mitochondrial genome fragmentation is correlated with increased rates of molecular evolution

**DOI:** 10.1371/journal.pgen.1011266

**Published:** 2024-05-03

**Authors:** Tomáš Najer, Jorge Doña, Aleš Buček, Andrew D. Sweet, Oldřich Sychra, Kevin P. Johnson

**Affiliations:** 1 Department of Veterinary Sciences, Faculty of Agrobiology, Food, and Natural Resources, Czech University of Life Sciences Prague, Prague, Czechia; 2 Illinois Natural History Survey, Prairie Research Institute, University of Illinois Urbana-Champaign, Champaign, Illinois, United States of America; 3 Departamento de Biología Animal, Universidad de Granada, Granada, Spain; 4 Biology Centre, Czech Academy of Sciences, České Budějovice, Czechia; 5 Okinawa Institute of Science & Technology Graduate University, Onna-son, Okinawa, Japan; 6 Department of Biological Sciences, Arkansas State University, Jonesboro, Arkansas, United States of America; 7 Department of Biology and Wildlife Diseases, Faculty of Veterinary Hygiene and Ecology, University of Veterinary Sciences Brno, Brno, Czechia; Colorado State University, UNITED STATES

## Abstract

While mitochondrial genome content and organization is quite diverse across all Eukaryotes, most bilaterian animal mitochondrial genomes (mitogenomes) exhibit highly conserved gene content and organisation, with genes typically encoded on a single circular chromosome. However, many species of parasitic lice (Insecta: Phthiraptera) are among the notable exceptions, having mitogenomes fragmented into multiple circular chromosomes. To better understand the process of mitogenome fragmentation, we conducted a large-scale genomic study of a major group of lice, Amblycera, with extensive taxon sampling. Analyses of the evolution of mitogenome structure across a phylogenomic tree of 90 samples from 53 genera revealed evidence for multiple independent origins of mitogenome fragmentation, some inferred to have occurred less than five million years ago. We leveraged these many independent origins of fragmentation to compare the rates of DNA substitution and gene rearrangement, specifically contrasting branches with fragmented and non-fragmented mitogenomes. We found that lineages with fragmented mitochondrial genomes had significantly higher rates of mitochondrial sequence evolution. In addition, lineages with fragmented mitochondrial genomes were more likely to have mitogenome gene rearrangements than those with single-chromosome mitochondrial genomes. By combining phylogenomics and mitochondrial genomics we provide a detailed portrait of mitogenome evolution across this group of insects with a remarkably unstable mitogenome structure, identifying processes of molecular evolution that are correlated with mitogenome fragmentation.

## Author summary

Mitochondria are organelles that play a key role in providing energy to most eukaryotic cells. The structure of the mitochondrial genome is conserved across most bilaterian animal groups, being a single circular chromosome containing 37 genes. Deviations from this structure are typically detrimental and, in humans, associated with diseases. However, in a few animal groups, the mitochondrial genome is fragmented into multiple circular chromosomes. In one group of insects, parasitic lice, fragmentation varies among species, with some having a complete circular genome and others having their mitochondrial genome fragmented in two or more smaller chromosomes. Here, we use whole genome sequencing data to analyze an unprecedented number of species from a diverse group of lice (Amblycera) that exhibits both single-chromosome and fragmented mitochondrial genomes to understand how this fragmentation evolved. We found that fragmentation evolved many times independently in this group and this fragmentation is correlated with faster rates of mitochondrial molecular evolution and with an increased frequency of gene rearrangement. We also provide evidence that the rate of mitochondrial genome fragmentation changes over time. Altogether, our combination of broad sampling and phylogenomic and comparative analyses provide new insights into the mechanisms and dynamics of mitochondrial genome fragmentation.

## Introduction

Mitochondria play a vital role in the metabolism of eukaryotic organisms, supplying cells with most of the energy necessary to function. Across Eukaryotes, the size, gene content, structure, and organization of the mitochondrial genome is incredibly diverse [1–3]. Mitochondrial genome (mitogenome) size can range from a few kilobases to several megabases [1–4]. Furthermore, although the majority of organisms have mitochondrial genes organized on circular chromosomes, linear chromosomes do occur across several eukaryotic lineages [1–3]. The number of chromosomes can also vary, with most organisms having their mitogenome on a single chromosome. However, fragmented mitogenomes have been reported for a variety of organisms, including plants [5–8], fungi [9], and a variety of animal groups. In animals, fragmented mitogenomes have been found in sponges [10, 11], cnidarians [12], nematodes (*Globodera*; [13]), thrips [14], and lice [15].

Despite this variability in the structure of mitogenomes across Eukaryotes, in some groups, the structure of the mitogenome is very stable across hundreds of millions of years. Most bilaterian animals, including insects, studied to date have their mitochondrial genetic information organised on a single circular chromosome between 12,000–18,000 base pairs long containing 37 genes [16]. Natural selection may promote conservation of this structure in insects. For example, in *Drosophila*, mutations that cause variation in this structure, such as mitogenome fragmentation, are highly deleterious [17,18]. In humans, any defect in this structure typically results in cell death [19], and is linked to degenerative diseases, ageing, and cancer [20,21].

However, in some insects, mitogenome structure deviates from the conserved, single chromosome state without having a notable effect on mitochondrial function. Particularly, within hemipteroid insects, mitogenome fragmentation appears to have evolved multiple times [14,15,22]. In some species in these lineages, the original single mitochondrial chromosome is fragmented into smaller chromosomes of variable size and number. Similar mitochondrial mutations have detrimental effects in humans [19,20] and *Drosophila* [17,18]. Therefore, understanding mitogenome fragmentation and reorganisation may provide significant insights for research on cell ageing or severe hereditary diseases.

Among the insect groups with fragmented mitogenomes, parasitic lice (Phthiraptera) show perhaps the broadest range of mitogenome structure. Several lineages of parasitic lice maintain mitogenomes on single circular chromosomes [23], while in others, the mitogenomes are highly fragmented and consist of up to 20 small circular chromosomes [24–26]. Recent evidence of heteroplasmy of mitochondrial genome structure (presence of multiple structural variants within a single individual) in some lice [15] suggests that these variants could be somehow involved in the process of fragmentation. Similar heteroplasmy has also been shown to evolve in experiments with nematodes [27].

Overall, lice offer an exceptional model to document the process of mitogenome fragmentation over time. Although it is well established that mitogenome structure is variable in parasitic lice, one important question is whether the rate of mitogenome fragmentation might change over time. To date, studies of mitogenome fragmentation in lice have primarily focused on mammalian lice (Trichodectera; [28]; Anoplura; [26]; Rhynchophthirina; [29]) and avian feather lice (Ischnocera; [15,24]). In the case of mammalian lice, the groups Anoplura (sucking lice), Rhynchophthirina (chewing lice), and Trichodectera (chewing lice) represent extreme instances of mitochondrial fragmentation. All members of these groups appear to have highly fragmented mitogenomes, with no cases of a single chromosome discovered in these groups to date. Thus, in Anoplura, Rhynchophthirina, and Trichodectera, mitochondrial fragmentation is highly phylogenetically conserved. However, in avian feather lice (Ischnocera), the opposite appears to be the case [15]. Within this group, mitogenome structure varies dramatically over the tree, with some species possessing a single mitochondrial chromosome while related genera are highly fragmented. This pattern makes it challenging to understand the process of stepwise fragmentation and whether there is phylogenetic conservation of mitogenome structure within Ischnocera. Given that mammalian lice and avian feather lice began diversifying around the same time [30], these differences in mitogenome structure suggest that the rate of fragmentation might change over time across all parasitic lice. Furthermore, other ecological factors such as differences in life history, host associations, effective population size, and nuclear/mitochondrial genome interactions may play a role in influencing mitochondrial fragmentation in lice.

Mitochondrial fragmentation in another major group of lice (Amblycera) is less well studied. One study of nine species in this group revealed five cases of single-chromosome mitogenomes and four of fragmented mitogenomes, which seem to have occurred multiple times [23]. A more recent study provided further evidence of multiple fragmentation events in two families of Amblycera (Laemobothriidae and Menoponidae; [31]). Given the potential for multiple independent origins of mitogenome fragmentation, Amblycera provides an excellent opportunity to study the evolutionary dynamics of mitochondrial fragmentation with greatly expanded sampling and comparative analyses.

**Fig 1:**
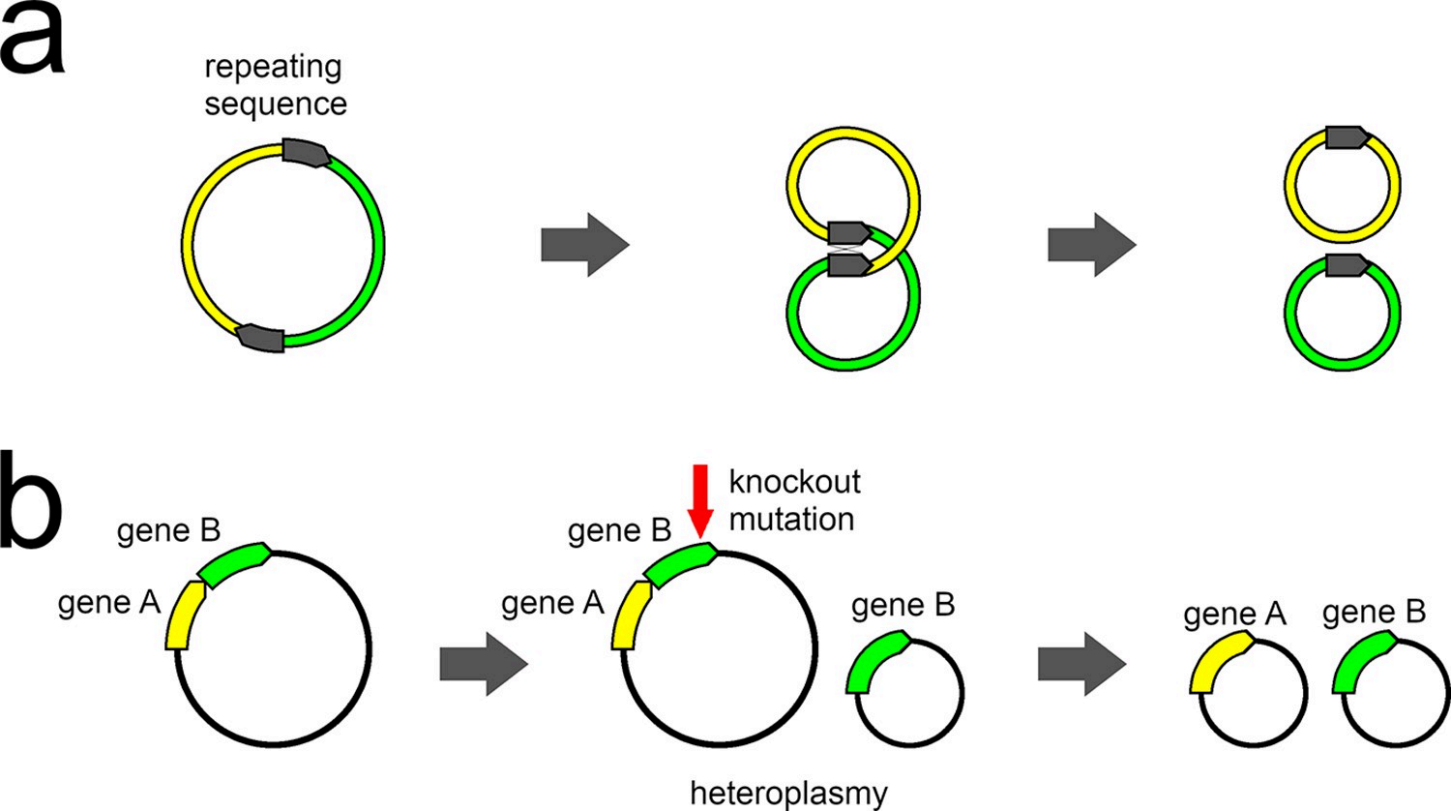
Illustration of mitochondrial genome fragmentation arising from a) recombination and b) heteroplasmy. In the case where a mitochondrial chromosome contains a repeating sequence (a, after [2]), the potential exists for the chromosome to recombine at this repeat. This recombination could lead to two separate circular chromosomes. In the case of heteroplasmy of mitogenome organization (b), one gene could be present on two different chromosomes (gene B). If a knockout mutation (*e*.*g*., deletion event) was to eliminate this gene on the original larger chromosome, natural selection would favour retaining the smaller partial chromosome, on which this gene still functions. This would lead to retention of both chromosomes in a situation where the genome is now fragmented.

**Fig 2:**
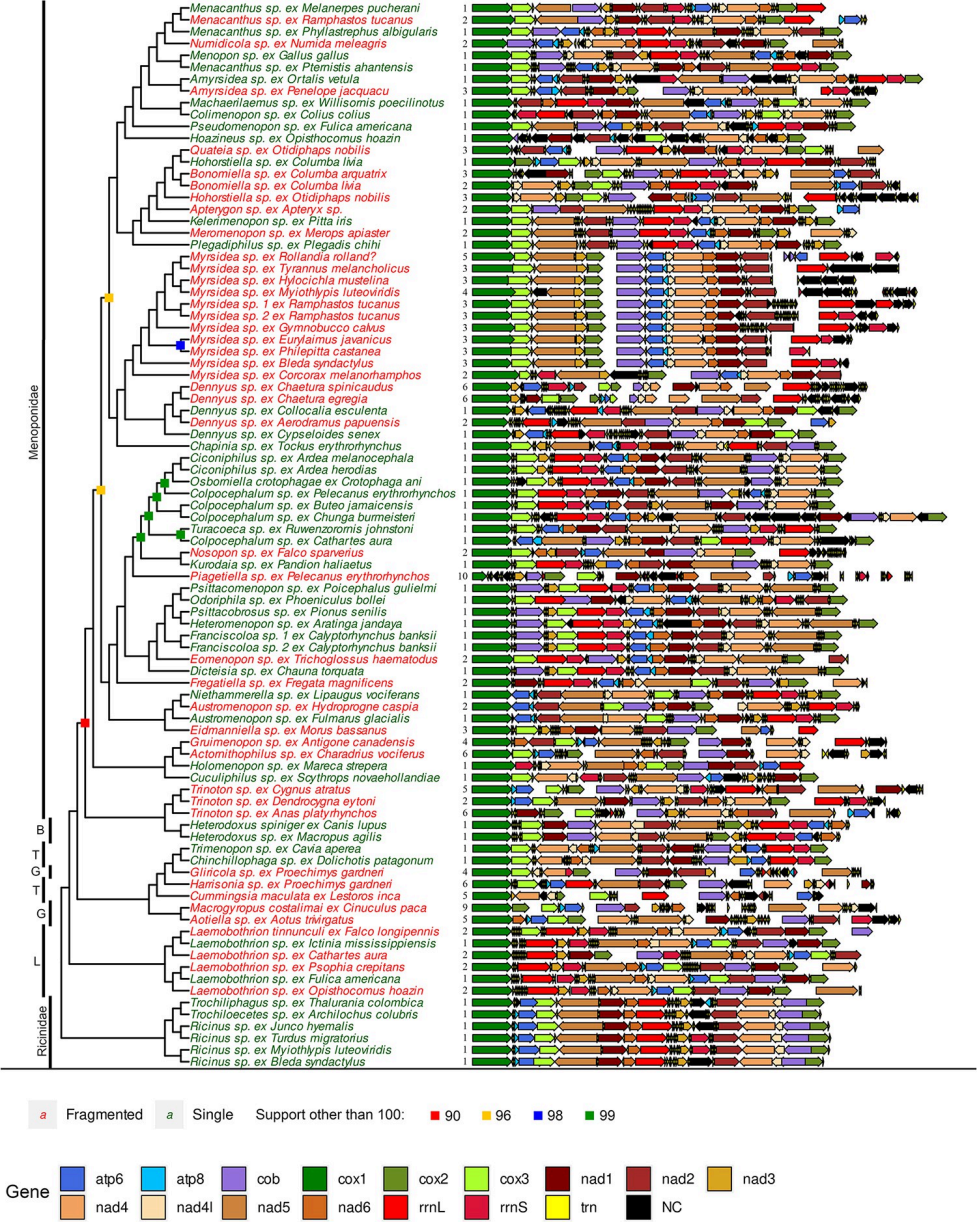
Phylogeny and pattern of mitogenome organisation across Amblycera. Phylogenetic tree based on concatenated maximum likelihood analysis of 2,395 target nuclear ortholog genes. Tree is drawn as a cladogram for readability. All branches supported by 100% ultrafast bootstrap, except those indicated with coloured symbols at node. Abbreviations of amblyceran families on left: B–Boopidae; T–Trimenoponidae; G–Gyropidae; L–Laemobothriidae. Diagrams are of assembled mitochondrial chromosomes for each taxon. Chromosomes have been arbitrarily linearized, starting with *cox1* for consistent directionality and to facilitate comparison. Numbers next to the mitogenome diagrams indicate numbers of fragments. Gaps between genes indicate separate mitochondrial fragments. Taxa with fragmented mitogenomes are highlighted in red, and those with single-chromosome mitogenomes in green.

There are two principal, although not necessarily mutually exclusive, hypotheses for the origin and mechanism of mitochondrial fragmentation (Fig 1). The first is that mitochondrial fragmentation is related to recombination of the mitochondrial genome [26,32]. This recombination could occur within a single large circular mitochondrial chromosome, particularly if there are repeat elements within the chromosome (Fig 1A; [2]). A second hypothesis for the origin of fragmentation suggests that initial heteroplasmy, in which a second partial copy of the mitochondrial genome is produced through deletion of a fragment between two mononucleotide repeats [25], triggers the process of fragmentation [15]. One possible mechanism for this deletion is stalled replication forks at hairpin-loop secondary structures caused by the two repeat regions [25]. It could also be that within-chromosome recombination at the mononucleotide repeat sequence triggered the initial heteroplasmy. Heteroplasmy of chromosome structure has been documented in lice with fragmented [15] and non-fragmented [25] mitogenomes. Extensive mitogenome fragmentation and heteroplasmy has also been reported for a parasitic plant [8]. Structural heteroplasmy results in two (or more) copies of several genes, and knockout mutations of one of the alternate copies could occur without being deleterious (Fig 1B). If this happens, both chromosomes that contain functioning copies of different genes would then be preserved by selection and then this process could iterate leading to increasing fragmentation.

Both of these hypotheses have implications for the process of mitochondrial gene rearrangements, which are also widely observed in lice [33]. A single mitochondrion often has multiple copies of the mitogenome [34], which could facilitate recombination among the copies. Thus, recombination in the mitochondrial genome could cause gene order rearrangements. In comparisons across several metazoan phyla, Feng et al. [32] observed that lineages that had cases of mitochondrial fragmentation also exhibited higher rates of gene rearrangement; however, the level of rearrangement within clades was not related to whether mitogenomes were fragmented or not. Under the heteroplasmy hypothesis, gene order could also change because it is expected that genes with any knockout mutations would eventually be deleted in one of the heteroplasmic copies, which would create novel gene boundaries. Thus, both hypotheses predict that lineages with fragmented mitochondrial genomes should have increased rates of gene rearrangement.

One pattern that might distinguish between the recombination model and heteroplasmy model is whether there are changes in the rate of fragmentation. In lineages where there is a complete mitochondrial genome, but also heteroplasmy of a partial genome, this model would predict a “chain reaction” in which fragmentation becomes much more likely. In contrast, in lineages that have no heteroplasmy, it is expected these lineages would be resistant to fragmentation. Under the recombination model, there is not necessarily an expectation that some lineages are more prone to recombination than others, and thus the rate of fragmentation might be predicted to be uniform across the tree.

Another topic of interest is whether fragmented mitogenomes have a higher rate of DNA base substitution than non-fragmented ones. Overall, mitochondria possess fewer repair mechanisms than nuclear genomes [35], making mitogenomes more susceptible to mutations. Another feature that has been observed to be associated with mitogenome fragmentation is an increase in the rate of substitution of mitochondrial DNA, possibly related to a higher underlying mutation rate. For example, in the plant genus *Silene*, Sloan et al. [5] found that some species in this genus have very high mitochondrial substitution rates compared to others, and this increased rate was associated with extensive fragmentation of the mitochondrial genome in these same species. Furthermore, Feng et al. [32] observed that mitochondrial substitution rates appeared to be faster in lineages that contained representatives with mitochondrial fragmentation. Because of their parasitic lifestyle, lice have undergone morphological simplifications and loss, which might result in overall relaxed selection across the genome [36]. This genome-wide relaxed selection would be predicted to result in reduced selection for mutation repair [37]. Thus, replicational and repair machinery might be similarly affected by this relaxed selection leading to correlations between changes in genome mutation rates and structural reorganization. Indeed, fragmentation in feather lice (Ischnocera) has been shown to be correlated with signals of relaxed selection in the form of dN/dS ratios [15]. In addition, comparisons of lineages of lice with single versus fragmented chromosomes living on the same host group showed that the lineage with fragmented mitogenomes had more mitochondrial divergence than the lineage without [38]. This increase could also occur if the smaller chromosomes of fragmented mitogenomes replicate more times per generation than non-fragmented mitogenomes.

The main goal of this study was to uncover the pattern of mitogenome fragmentation in a major group of lice, Amblycera, with unprecedented taxonomic and temporal resolution. Using genomic sequencing reads, we assembled the mitochondrial genomes of 90 samples of Amblycera, representing 53 genera and 89 species, and reconstructed a dated phylogenomic tree based on over 2,000 nuclear single-copy ortholog genes. With these data, we traced the phylogenetic pattern of the fragmentation across Amblycera, inferring the number of transitions from non-fragmented to fragmented mitogenomes and shedding light on the dynamics of this process. Furthermore, we used the evolutionary changes in fragmentation to estimate whether the rate of fragmentation changes across the tree. We also tested two key predictions of both models of mitogenome fragmentation by examining the correlation of both gene rearrangement and apparent substitution rate (i.e., branch length) with fragmentation.

## Results

### Phylogenomics clarifies phylogenetic relationships within Amblycera, uncovering paraphyly of some families

Our phylogenomic analysis based on 2395 nuclear orthologs provided a well-resolved and supported tree for Amblycera. Both concatenated and coalescent analyses strongly support the monophyly of Amblycera (100% support; S1 and S2 Figs). There were only a few differences between the concatenated and coalescent trees, mostly involving changes in the relationships among some families (S1–S3 Figs). Within Amblycera (Figs 2, S1, and S2), our phylogenomic tree confirms the monophyly of the families Ricinidae and Laemobothriidae (Fig 2). Concerning the family Boopidae, we examined only two samples of one genus, so although they are sister taxa, fully testing the monophyly of this family would benefit from more taxon sampling. Both the concatenated and coalescent trees suggest that the families Gyropidae and Trimenoponidae are paraphyletic. These two families intertwine to form a single monophyletic clade of lice (Figs 2, S1, and S2) parasitising Neotropical mammals, primarily rodents and marsupials [39]. The concatenated (S1 Fig) and coalescent (S2 Fig) trees differ in the positions of the genus *Trinoton* and the family Boopiidae. In the concatenated tree (S1 Fig), the genus *Trinoton* is sister to Boopiidae, rendering Menoponidae paraphyletic. However, in the coalescent tree (S2 Fig), Boopiidae is a sister to all other Amblycera, while *Trinoton* is a sister to the remainder of Menoponidae, making this latter family monophyletic. In both trees, the remainder of Menoponidae collectively forms a large monophyletic clade (Figs 2, S1, and S2). At the generic level, our data also suggest some genera may not be monophyletic, including *Austromenopon*, *Menacanthus*, *Hohorstiella*, *Colpocephalum*, and *Ricinus* (Figs 2, S1, and S2).

### Mitochondrial genome fragmentation is widespread among Amblycera

We assembled mitogenomes from 90 samples across 53 genera (89 species) of Amblycera (S1 Table), finding evidence for circularity in most chromosomes (186 out of 197; S2 and S3 Tables). We recognize that other physical structures, such as linear concatemers or circularly permutable linear molecules [2], can assemble as circles using read assembly techniques. While the assemblies of the lice in our study are consistent with fragmented minicircular organization, this architecture is not yet directly confirmed by physical evidence (such as Southern blot or electron microscopy) in these species. Thus, our assemblies should be considered “circular-mapping”, given this caveat.

Even though we have evidence of circularity from read-based analyses (Simple-Circularise and AWA), we also examined contigs for sequences shared between contigs, which could potentially be responsible for assembly artifacts. We found only three species in which there was a sequence longer than 150 bp (the length of one read) shared between chromosomes: *Dennyus* sp. ex *Chaetura egregia*, *Trinoton* sp. ex *Cygnus atratus*, and *Piagetiella* sp. In the cases of *Dennyus* sp. and *Trinoton* sp., we were able to find evidence of paired-end reads that mapped to both ends of a contig or single reads that mapped to both ends of a contig. In the case of *Piagetiella* sp., we were not able to find reads that would strongly link the two ends of a contig. The mitogenome of *Piagetiella* sp. was particularly difficult to assemble, and some contigs did not pass AWA circularity test or had some genes cut away by the AWA procedure (S2 Table). Generally, mitogenomes in lice that are a single circular chromosome are straightforward to assemble. Thus, we suspect that the mitogenome of *Piagetiella* sp. is fragmented but was difficult to assemble because of these repeat elements. Given these concerns with the assembly of *Piagetiella* sp., we also repeated any impacted analyses under the assumption that it was non-fragmented, and none of these analyses changed in significance or in the overall conclusions.

Mononucleotide repeats have been suggested to be potentially important in the process of mitogenome fragmentation [25]. We found only nine samples with instances of mononucleotide repeats of 18 bp or more. All of these were A or T repeats, often in the same chromosome (S4 Table). Of these nine samples, two had fragmented chromosomes, and seven had non-fragmented chromosomes. These repeat regions could potentially facilitate fragmentation, but they do not appear to be widespread in our study.

**Fig 3:**
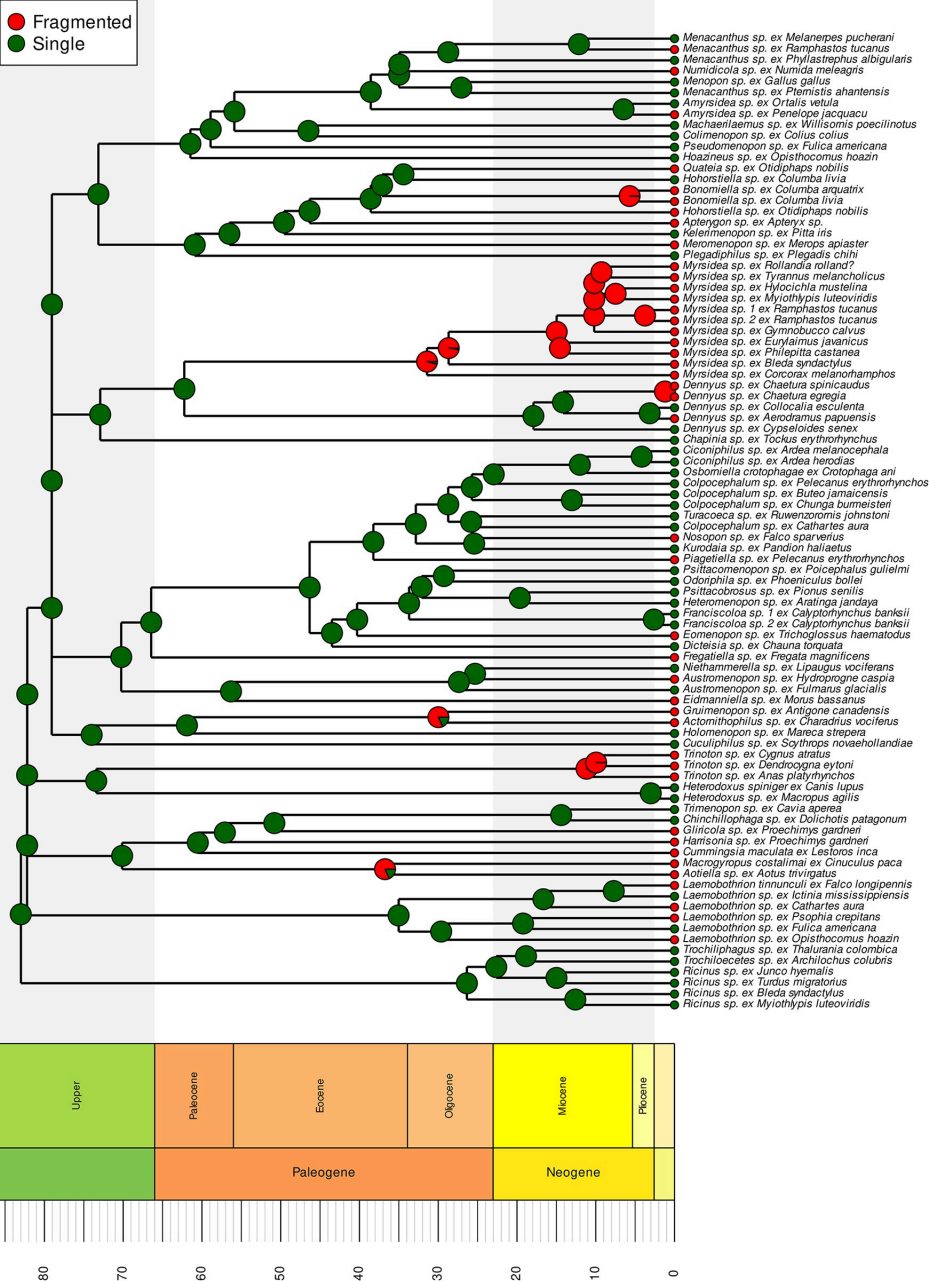
Dated phylogenomic tree with ancestral state reconstruction of mitogenome evolution in Amblycera under irreversible model. Circles at the tips indicate mitogenome structure (single-chromosome versus fragmented). Pie charts at the nodes show the frequency distribution of reconstructed ancestral state after 1000 simulations of stochastic character mapping using an irreversible fragmentation model (USR). Time scale at bottom in millions of years ago (Mya).

Across our assemblies, we were able to identify all protein-coding genes in the majority of samples (78; S2 and S5–S17 Tables). Given the above caveats regarding read-based assembly, many species were inferred to have single circular mitochondrial chromosomes, but there were also many instances of mitogenome fragmentation (Figs 3 and S4), and some of these fragmentation events occurred over short timescales (< 5 Mya; Figs 3 and S4). We found significant phylogenetic signal for mitogenome fragmentation within Amblycera (D-value = 0.281, P = 0.001; S5 Fig), consistent with a Brownian motion model (P = 0.18). Among the six currently recognised families of Amblycera (Boopiidae, Gyropidae, Laemobothriidae, Menoponidae, Ricinidae, Trimenoponidae; [39]), all except Gyropidae contain samples with single-chromosome mitogenomes (Fig 2). In particular, all samples from Ricinidae (6 spp.) and Boopiidae (2 spp.) possess single-chromosome mitochondrial genomes with largely consistent gene orders. Notably, most members of Ricinidae, the sister group to all other Amblycera, retain the gene order *atp8-atp6-cox3*, also seen in free-living book lice (*Liposcelis*; [22]). The largest family, Menoponidae, which exclusively inhabits birds, displays a high degree of mitogenome structural variation, ranging from single-chromosome to highly fragmented across multiple clades (Fig 2). The families Gyropidae and Trimenoponidae, both exclusive to mammals, form a clade in our analysis, yet they are not mutually monophyletic. Within these families, mitogenome structure varies from single-chromosome (in *Trimenopon* and *Chinchillophaga*) to highly fragmented with up to nine mitochondrial chromosomes (in *Macrogyropus*).

Our samples included multiple species within several amblyceran genera, allowing us to investigate variation in mitogenome structure among closely related taxa. In particular, we observed transitions from single-chromosome to fragmented mitogenomes within the genera *Menacanthus*, *Amyrsidea*, *Dennyus*, *Austromenopon*, and *Laemobothrion* (Fig 2). Moreover, we observed changes in the number of fragments between species within *Myrsidea* and *Trinoton* (Fig 2). Consistent with previous studies (e.g., [15,33]), we found that gene order (Fig 2 and S2 Table), even for single-chromosome genomes, was massively rearranged between lineages. However, in a few instances, the gene order remained stable among single-chromosome taxa, particularly within Ricinidae and Laemobothriidae (Fig 2 and S2 Table). Our data suggest a possible trend towards increasing fragmentation within the genus *Myrsidea*. The sister taxon (*Myrsidea* sp. ex *Corcorax melanorhamphos*) to the rest of *Myrsidea* possesses two chromosomes, while the remaining species of *Myrsidea* have three chromosomes, and some more derived species exhibit even four or five chromosomes. This pattern suggests a gradual increase in the number of fragments over the course of the evolution of *Myrsidea* (Fig 2).

It is often assumed that once mitogenome fragmentation occurs, it is irreversible (e.g., [15]). Indeed, we found several highly supported cases where an ancestral state of a single chromosome transitions to a fragmented state (e.g., *Menacanthus*, *Nosopon*, and *Eomenopon*). Despite this, our analysis did not reject the equal rates (ER) model for the evolution of mitochondrial fragmentation across Amblycera, and it was indeed the best-fitting model for this analysis (AIC = 109.8825, AICc = 109.928, AIC weight = 0.4197; S18 Table). The ER model suggests nearly equal likelihoods for ancestral states of single versus fragmented mitogenomes in Amblycera (S4 Fig). Under this scenario, there are only three instances in the phylogenetic tree where a likely transition (>75% relative likelihood) from fragmented to single is inferred (*Hohorstiella* and two cases in *Laemobothrion*). However, for most taxa, the likelihood of the ancestral state does not strongly favour either state. Similarly, parsimony reconstruction was also ambiguous as to the mitogenome structure in the common ancestor of Amblycera (S6 Fig). However, under parsimony, many internal nodes were reconstructed to be single-chromosome in structure, with many transitions to fragmented mitogenomes. One caveat is that our analysis does not take into account the genome organisation of more distant outgroups among free-living Psocodea, the overwhelming majority of which have single mitochondrial chromosomes like most other insects. Therefore, it is likely that the ancestral state for Amblycera would be a single mitochondrial chromosome (Fig 3). The reconstruction that assumes irreversibility of fragmentation (AIC = 111.5266, AICc = 111.6646, AIC weight = 0.0040; Fig 3 and S18 Table) suggests at least 27 transitions from single-chromosome to fragmented mitochondrial genomes. While it remains to be seen if any definitive case of fragmentation reversal (i.e., from fragmented to single) exists, further sampling within *Hohorstiella* and *Laemobothrion* could shed more light on this matter. Overall, fragmentation extensively varies across Amblycera, yet not to such an extent that it obscures overall evolutionary patterns.

### Rate of mitogenome fragmentation across Amblycera

**Fig 4:**
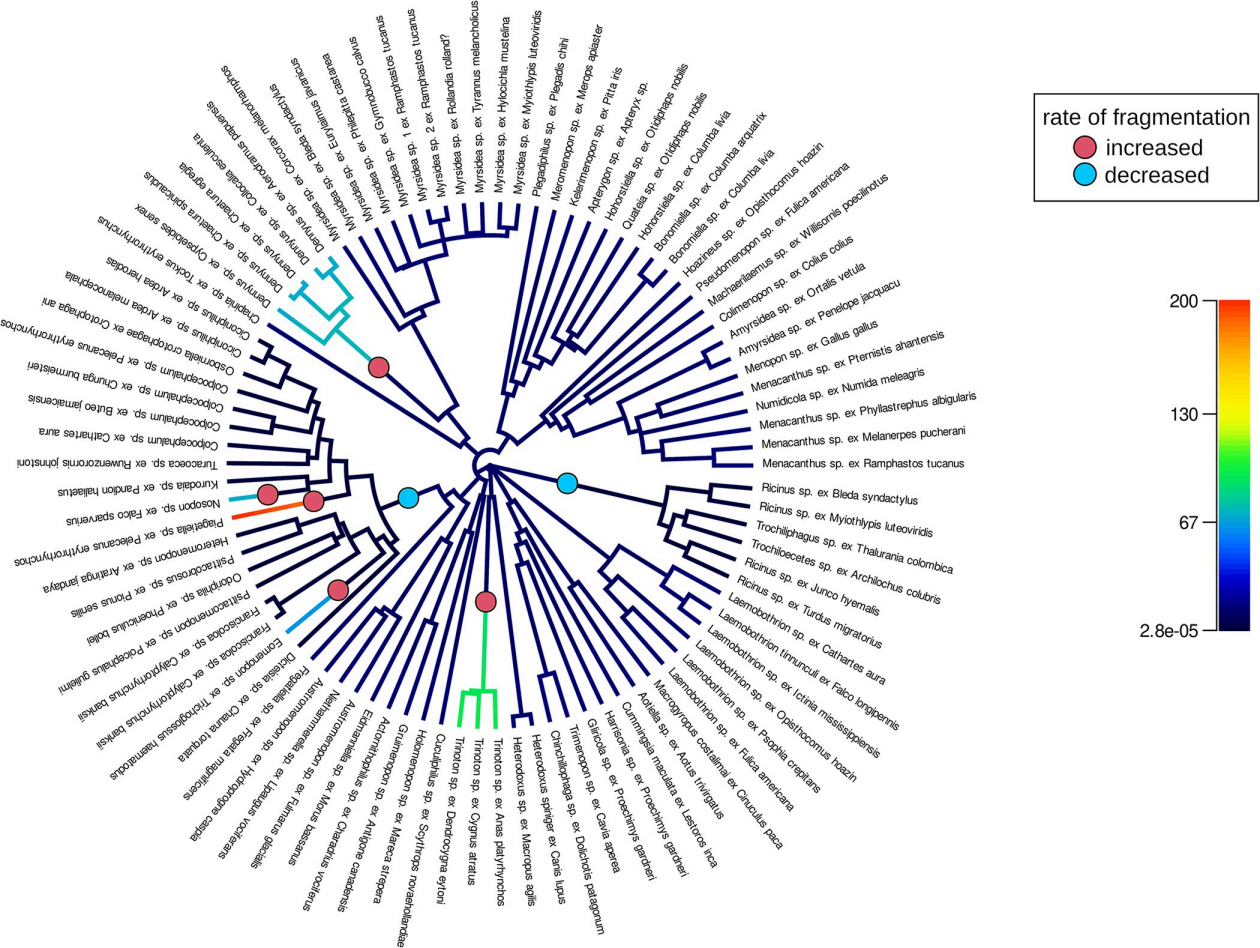
Changes in fragmentation rate in mitogenomes of Amblycera. Tree based on concatenated maximum likelihood analysis of 2,395 target nuclear ortholog genes. Tree drawn as circular phylogram (outgroup root not shown). Circles at the branches indicate rate shift events included in the optimal reconstruction (with maximum *a posteriori* probability). Significant rate increases indicated with red circles, and rate decreases with blue circles. Overall reconstructed rate of mitogenome fragmentation on each branch shown with color from low rate (dark blue) to high rate (red) as indicated by the scale bar.

The BAMM analyses provided evidence of significant changes in the rate of fragmentation over the amblyceran tree. The most probable solution in the analysis of the reconstructed rate of fragmentation (Fig 4) suggests seven rate shift events. In two cases (family Ricinidae and common ancestor of a group containing the *Colpocephalum* complex), the rate of fragmentation slowed down. The five cases in which the rate of fragmentation increased involved single genera (*Trinoton* and *Dennyus*) or terminal taxa (*Eomenopon*, *Piagetiella*, and *Nosopon*). The nine most frequent solutions of the analysis (S7 Fig) suggest between 5–8 rate shift events mainly positioned on the same branches as in the most probable solution.

This variation suggests that the rate of fragmentation might change across the tree. In some cases, mitogenome structure is stable for a long period of time, while in others, it appears to change rapidly. In Ricinidae, both mitogenome structure and gene order remained stable for at least around 30 million years (Figs 2, 3, and S4). In contrast, over this same timeframe within the family Laemobothriidae, there are multiple transitions in mitogenome structure (Figs 2, 3 and S4). These observations are borne out by detection of significant shifts in the rate of fragmentation over the tree. Specifically, the rate of fragmentation in Ricinidae is inferred to have decreased compared to the rest of the tree (Fig 4). Increases in the rate of fragmentation are recovered for *Dennyus* and *Piagetiella* (mentioned above), as well as on three other branches (Fig 4). In the genus *Myrsidea*, a relatively consistent three-fragment structure remained unchanged for an extended period across much of the diversification of the genus (33–10 Mya). However, in more recently diverged lineages within *Myrsidea*, additional fragmentation took place (Figs 3 and S4), but all these changes occurred at a similar rate to the overall rate across the tree (Fig 4). Given the notable diversity of *Myrsidea*, containing nearly 400 described species [39], it is likely that novel mitogenome configurations may be discovered in other species within this genus. In the case of *Dennyus*, the closest relative to *Myrsidea* (Figs 2–4 and S1–S4), it seems that its ancestor may have possessed a single-chromosome mitogenome which underwent a recent fragmentation event (within 2–5 My), resulting in six small minicircles (Figs 1–3 and S4), and this likely occurred at a faster rate than changes over the rest of the tree. However, intermediate states of fragmentation cannot be completely ruled out. Even though evidence suggests that mitogenome structure typically remains stable within a given species (e.g., *Columbicola passerinae* [15], *Franciscoloa* sp. ex *Calyptorhynchus banksii* [this study]), we uncovered instances where considerable variation can occur within a single genus (e.g., *Laemobothrion*, *Menacanthus*), sometimes over less than five million years (e.g., *Dennyus*, Figs 3 and S4).

### Comparison of substitution rates and gene rearrangements

**Fig 5:**
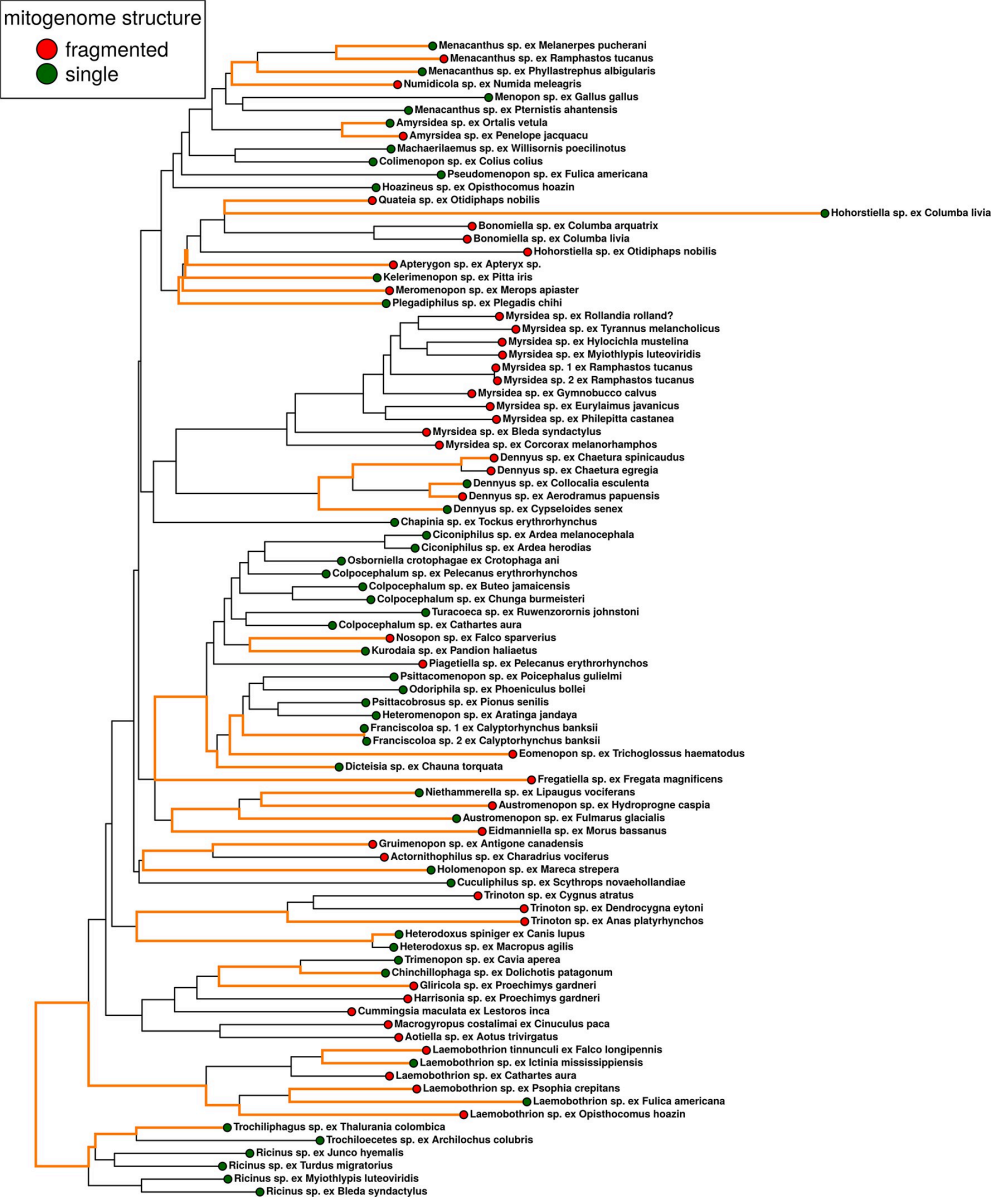
Pairs of Amblycera selected for comparison of branch lengths. Circles at the tips indicate mitogenome structure (single-chromosome versus fragmented), and samples compared within a pair are connected with orange line. Branch lengths computed from mitochondrial alignment, topology preserved from the nuclear concatenated tree.

We compared the lengths of both mitochondrial and nuclear branches in 19 Independent Matched Pair Comparisons within Amblycera (Fig 5 and S19 Table) between fragmented and non-fragmented lineages. For mitochondrial branch lengths, lineages with fragmented mitogenomes had longer branch lengths in 14 of 19 pairs (Sign test *P* = 0.039; Fig 5 and S19 Table). This result indicates mitochondrial protein-coding genes evolve significantly faster in lice with fragmented mitogenomes compared to those with non-fragmented genomes. For nuclear branch lengths, lineages with fragmented mitogenomes had longer branches in 13 of 19 pairs (Sign test *P* = 0.108; S8 Fig and S19 Table).

Similarly, we compared gene rearrangements between fragmented and non-fragmented taxa for 19 Independent Matched Pair Comparisons within Amblycera (slightly different than the pairs for branch lengths comparison; S8 Fig and S20 Table). In 14 of 19 pairs, the fragmented mitochondrial genomes were more rearranged, compared to the reconstructed common ancestor (S21 Table), than non-fragmented genomes (Sign test *P* = 0.039). Although we did not manage to identify all tRNA genes in all the mitogenomes, the differences in gene rearrangements were so large (S20 Table) that even if we hypothetically placed them in the same positions as in their sisters, it would not affect the categorisation of which mitogenome is more rearranged. Consistent with Feng et al. [32] and Sweet et al. [15], the rRNA and tRNA genes were responsible for most gene rearrangements, having unstable positions even between closely related species (e.g., genera *Amyrsidea*, *Bonomiella*; S2 Table).

### Fragmented mitogenomes of Amblycera are on average less AT biased

Comparison of the base composition (AT percentage) of single-chromosome mitochondrial genomes to fragmented genomes indicates that the AT composition of single-chromosome genomes is significantly higher overall (S9 Fig and S22 Table). Although the AT content of fourfold degenerate sites is higher than that of other positions (S10 Fig), the AT content of fourfold degenerate sites from single-chromosome mitogenomes is notably higher than that of fourfold sites from fragmented mitogenomes (S11 Fig). The decrease in AT bias in fragmented mitogenomes is also observed at all other positions (S11 Fig). These findings align with previous studies [15,23], reinforcing the hypothesis of differing mutational or selective biases in fragmented versus single-chromosome mitochondrial genomes. The differences in base composition across different protein-coding genes (S12 Fig and S5–S17 and S23 Tables) can be primarily attributed to disparities between fourfold degenerate sites and other partitions. In general, fourfold degenerate sites more likely reflect mutational biases rather than direct selection. These differences are especially evident in *cox1-3* and *cob* genes, which also exhibit the most conserved gene arrangements (Fig 2). We did not observe a significant correlation between chromosome length and AT content in fragmented mitogenomes.

## Discussion

An important assumption of our study is that we can successfully identify instances of mitogenome fragmentation from Illumina read-based assembly techniques. Generally, the physical structure of mitogenomes cannot confidently be determined from assembly or PCR based techniques alone, because of artifacts in these approaches [2]. Physical (Southern blot or electron microscopy) methods are needed to fully infer fragmentation and the circular nature of these chromosomes. The most well studied louse with respect to fragmented mitogenome organization is the human louse (*Pediculus humanus*), whose mitogenome is organised into 18 minicircular chromosomes [26]. This state was initially discovered from assembly of Sanger shotgun sequencing reads, but also more fully confirmed by Southern blot analysis [26]. PCR amplification and sequencing of these minicircular chromosomes have provided additional evidence for minicircular mitogenome fragmentation in *Pediculus humanus*. A follow-up study [24] applied computational assembly techniques with short Illumina reads to *P*. *humanus* and successfully assembled the fragmented mitogenome of this species with evidence of circularized chromosomes. Further studies using both PCR and read assembly based techniques across a diversity of lice have provided evidence for the presence of mitogenome fragmentation and minicircular chromosomes in a wide variety of species [25,29,31,40]. The results from *Pediculus* support the idea that fragmented mitogenomes of other lice are also likely to be minicircular in organisation, but it would be useful to provide physical evidence in species that are less fragmented than *Pediculus*. However, taken together, these prior studies indicate that read based assembly techniques can potentially be successful at assembling both fragmented and non-fragmented mitogenomes in lice and provide a foundation for further study of louse mitogenome organisation using physical techniques.

By leveraging an extensive dataset of 90 samples of lice (53 genera, 89 total species) in the parvorder Amblycera, and integrating mitogenome assembly with nuclear phylogenomics, we gained substantial insights into mitogenome fragmentation within a diverse group of parasitic lice. We observed that mitogenomes undergo fragmentation numerous times (potentially 27 or more) across Amblycera, with some fragmentation events occurring as recently as a few million years ago. In various genera represented by more than one sample, transitions from a single chromosome to a fragmented mitogenome (e.g., *Dennyus*, *Laemobothrion*, *Menacanthus*), as well as increasing fragmentation into a larger number of fragments (e.g., *Myrsidea*, *Trinoton*) were apparent. Despite the occurrence of fragmentation events over many branches in the tree, the fragmentation process shows some evidence of phylogenetic conservation. Certain louse groups appear to fragment more frequently and rapidly than others, and some broad clades are either entirely fragmented or entirely single-chromosome. Among the six recognised amblyceran families, fragmentation has evolved in at least four of these, although the monophyly of some families is not supported. The results of reconstruction of the mitogenome structure in the common ancestor of Amblycera from the current data depends heavily on the model used (i.e., whether reversal of fragmentation is permitted in the model or not). However, given the general conservation of mitogenome organisation across insects and in the common ancestor of Amblycera and free-living bark lice, it is highly probable that the common ancestor of Amblycera had a single mitogenome chromosome, with several transitions to fragmented mitogenomes occurring over time. Although transitions from a single chromosome to fragmented chromosomes seem the most likely, the hypothetical merging of chromosomes [41] through homologous and non-homologous mitochondrial recombination [42] has been suggested as a possible mechanism by which fragmented mitogenomes might merge back into a single chromosome [2,6]. However, even under the ER model, most changes are reconstructed to be from single to fragmented chromosomes.

In terms of the size of mitogenome fragments, we observed two primary patterns: 1) a small fragment encompassing a few genes splits from an original singe-chromosome mitogenome (e.g., *Laemobothrion*; *Numidicola*; *Menacanthus*; *Meromenopon;* [23]); 2) a single-chromosome mitogenome disintegrates into multiple smaller fragments of more similar sizes (e.g., *Myrsidea*, *Dennyus*; a similar pattern observed in Ischnocera, [15]). These two patterns are not mutually exclusive, and they can co-occur within a single individual (e.g., *Quateia*). In addition, numerous mitogenomes possess a state intermediate to these extremes. Furthermore, the genes involved, fragment sizes, and number of fragments seem to display considerable variation among different fragmentation events. Some studies [25,26] hypothesised that mitogenomes comprised of numerous small minicircles would evolve incrementally from a few larger fragments rather than directly from a single-chromosome mitogenome. However, the mitogenomes of the genus *Dennyus* suggest a possible “big bang” fragmentation event, when multiple small fragments appear at relatively fast rate, might indeed occur in lice, leading to a transition from a single to several mitochondrial chromosomes. Additional sampling within these genera might, however, uncover extant lineages with intermediate fragment numbers.

The BAMM MCMC analysis revealed significant shifts in the rate of mitogenome fragmentation across the tree, with some lineages being highly stable and others changing rapidly. Variation in the rate of fragmentation is a prediction of the heteroplasmy model of mitogenome fragmentation. In this case, lineages with chromosomal heteroplasmy would be prone to gene inactivation on one of the chromosome copies [8], which would then require the retention of the other copy of the chromosome [15]. This cycle would facilitate further gene deletion (i.e., fragmentation) within each chromosome. Thus, we would expect lineages with a heteroplasmic chromosomal state to fragment faster than lineages without heteroplasmy, which would be resistant to fragmentation. However, we did not detect any obvious cases of heteroplasmy in our data set, albeit it can be difficult to detect. The recombination model does not necessarily predict variation in the rate of fragmentation, but rather that fragmentation should be related to opportunities for recombination, possibly associated with the presence of repeat elements in the mitogenome [2,6]. However, if the presence of repeat elements varies across the tree, the recombination model might also predict variation in the rate of fragmentation.

In addition to variation in the rate of fragmentation, we also found variation in the rate of mitochondrial base substitution in protein-coding genes across the tree, perhaps reflecting underlying mutation rate. Specifically, we found a correlation between whether a genome is fragmented or not and overall substitution rate (as measured by branch length, and which is directly proportional to mutation rate, all else being equal), which might support a link between mutation rate and likelihood of fragmentation. Taxa with fragmented genomes tended to have longer branches in the tree, reflecting overall substitution rate, compared to paired comparisons with taxa with non-fragmented genomes. Such a linkage has also been reported in plants [5]. It is unclear, however, whether variation in mutation rate might cause fragmentation, or whether fragmentation causes an increased mutation rate, or perhaps they are both correlated with some other underlying factor.

We also found significant evidence that taxa with fragmented mitogenomes have more gene rearrangements than their non-fragmented relatives in paired comparisons. This finding is consistent with both the heteroplasmy and recombination models of mitogenome fragmentation. However, it is also the case that even non-fragmented mitogenomes within Amblycera have many gene rearrangements compared to the ancestral insect [33]. It seems unlikely that these rearrangements could be caused by heteroplasmy alone. Recombination with heteroplasmic chromosome copies could potentially facilitate these rearrangements, so it may be that both heteroplasmy and recombination are important together to fully understand mitogenome evolution in lice.

Concerning the initial drivers of fragmentation in lice, genome-wide relaxed selection has been posited as a possible mechanism for triggering changes in molecular evolution, including mitogenome organisation and mutation rates [25]. Indeed, Yu et al. [8] describe a highly fragmented mitochondrial genome of a parasitic plant. Although relaxed selection is typically associated with parasitism and reduced morphology, it does not account for why other arthropod parasites (e.g., fleas, ticks) lack fragmented mitogenomes or why fragmented mitogenomes also appear in free-living animals. We did, however, find a trend that amblyceran lineages with fragmented mitogenomes also tended to have higher rates of substitution for nuclear genes (13 of 19 cases), although this was not statistically significant (*P* = 0.108). If this trend holds with additional sampling in the future, it might suggest that overall relaxed selection for mutation repair might be driving mitogenome fragmentation. Indeed, one hypothesis [25,43] for the extremely fragmented mitogenomes of Anoplura is the absence of *mtSSB* in this group, as is the case of *Pediculus humanus* (Anoplura) [43]. The *mtSSB* gene is a nuclear gene that produces a protein (mitochondrial single-stranded DNA binding protein) targeted to the mitochondrion that stabilises mitochondrial DNA during the single-stranded replication stage. Lack of *mtSSB* could also explain the higher rate of DNA substitution in lineages with fragmented mitogenomes, because the *mtSSB* protein protects single-stranded DNA from mutations during replication [44]. Whether *mtSSB* is present in some lice and not others is currently unknown. A parasitic plant with a highly fragmented mitogenome and extensive heteroplasmy also appears to lack mitochondrion targeted DNA-RRR genes in its nuclear transcriptome [8]. Therefore, this general hypothesis presents an avenue for further research. Overall, our dense taxon sampling within a single clade of lice provided an unprecedented number of phylogenetically independent comparisons of fragmented and non-fragmented mitogenomes to test important patterns of molecular evolution under this unusual phenomenon.

## Material & methods

### Ethical statement

Research on animals was conducted according to University of Illinois Institutional Animal Care and Use Committee protocols 10119, 13121, 15212.

### Sequence data

We analysed the mitogenomes of 90 single specimens of amblyceran lice, a sample including all families, 53 genera, 89 species, the majority of host groups, and most biogeographic regions across which Amblycera occur. Of these, 84 were newly sequenced for this study. We photographed individual specimens as vouchers and extracted total genomic DNA using a Qiagen QIAamp Micro Kit, with a 48-hour initial incubation [45]. We then prepared libraries from these extractions with a Hyper library kit (Kapa Biosystems) and sequenced them on an Illumina NovaSeq 6000 with 150 bp paired-end reads [45]. We identified the vouchers to the genus level based on morphology using illustrations and keys [39,46]. We also included data from six additional amblyceran species analysed by Sweet et al. [23] from NCBI SRA [30]. We conducted a quality check on the raw data from all 90 samples using FastQC v0.11.9 (https://www.bioinformatics.babraham.ac.uk/projects/fastqc/) and trimmed the reads using BBDuK from the BBMap package (https://sourceforge.net/projects/bbmap/, setup ktrim = r k = 23 mink = 7 hdist = 1 tpe tbo maq = 10 qtrim = rl trimq = 35 minlength = 35). We trimmed adapters automatically and manually trimmed the 5’ and 3’ ends using the forcetrim = argument.

### Mitochondrial genome assembly and annotation

To avoid excessive coverage of the mitochondrial genome (which can lead to assembly errors) and decrease the computational demand, each sequence read library was subsampled for 2 million reads of Read1 and the corresponding Read2 (4 million total reads). The mitochondrial genomes were first assembled using MitoFinder v1.4.1 [47] with MetaSPAdes as the assembler, which is a de novo assembly algorithm designed for assembling multiple genomes within a pool of mixed reads. From the MetaSPAdes results, contigs similar to the reference (i.e., concatenated nucleotide sequences of previously published mitogenomes; [23,28]) were selected using TCSF v2.7.1 [48] with default parameters. From the TCSF results, contigs with high coverage (typically exceeding 100X) and a cumulative length of at least 15 kb were manually selected. These contigs were tested for circularity in Simple-Circularise (https://github.com/Kzra/Simple-Circularise) and AWA [49], searching for *k*-mers up to 40 bp long, mapped on full trimmed reads without subsampling. AWA is a method that splits a contig between two ends with matching *k*-mers, and then splices the 5’ end of the contig to be adjacent to the 3’ end. Reads are then mapped to this spliced contig and sequence similarity, coverage and average alignment score are calculated to derive a modified per-position coverage value, termed connectivity. Connectivity evaluates the support for an adjacency of two nucleotides with a short sequence fragment. This connectivity value is particularly relevant at the splice point, and high connectivity supports the inference of circularity. Manual inspection of gene overlap and sequence similarity, along with the AWA results, further validated circularity (S3 Table). Because repeat sequences can result in assembly artifacts, we searched each assembly for sequences longer than 150 bp (i.e., the length of one read) shared between contigs using Jalview [50]. Prior studies have also indicated that mononucleotide repeats may be hotspots for triggering mitogenome fragmentation [25]. Thus, we also searched the assembled mitogenomes for mononucleotide repeats of 18 bp or more.

The total fraction of reads derived from the mitogenome varied between samples. In some cases, a subsample of 4 million total reads did not appear to provide sufficient coverage of the mitogenome for a complete assembly. Thus, if the assembly failed to provide circular contigs encompassing all mitochondrial genes, the assembly procedure was repeated with subsampling increased to 8 million, and then 20 million total reads if necessary, to obtain sufficient coverage. For subsamples that produced successfully circularized contigs, we also tested increasing the number of reads for these libraries. In all of these cases (52), the assemblies with the higher subsample of reads were identical to those obtained with the lower subsample, suggesting that incrementally increasing the subsample of reads is an appropriate strategy both to avoid excessive coverage, but also to obtain sufficient coverage to produce accurate assemblies. Using MITOS2 [51], we annotated the contigs and identified genic regions overlapping the 3’ and 5’ ends. To verify annotation of protein-coding genes (PCGs) and find PCGs not located by MITOS2, we manually searched open reading frames (ORFs) identified by ORF Finder, part of Sequence Manipulation Suite, [52], and visually inspected ORFs in Jalview v2.11.2.0 [50].

### Phylogenomics, dating, and ancestral state reconstruction

For phylogenomic analysis of Amblycera, we used whole genome sequencing data of 90 amblyceran samples and 29 outgroup taxa from Ischnocera, Trichodectera, Rhynchophthirina, Anoplura, and free-living Psocodea [53]. For this analysis, reads were trimmed for adaptors and quality (phred score < 30) using *fastp* v0.20.1 [54] and converted to aTRAM 2.1 [55] databases. We assembled a target set of 2395 single-copy ortholog PCGs from the human head louse (*Pediculus humanus*) for each genomic library using aTRAM, using *tblastn* and the AbySS assembler (iterations = 3 and max-target-seqs = 3000). The resulting sequences were stitched using Exonerate with the reference protein-coding sequences, exons aligned, and genes trimmed using established tools and parameters [44,56]. We conducted a phylogenomic analysis on the concatenated alignment (81.3% matrix completeness; S24 Table) under maximum likelihood and using the GTR+G model in IQ-TREE 2 v2.1.2 [57]. Support was estimated using ultrafast bootstrapping (UFBoot2; [58]) with 1000 replicates. We also performed a coalescent analysis in ASTRAL-III v5.7.4 [59] to account for gene-tree/species-tree discordance. Separate gene trees were inferred using IQ-TREE, based on the optimal models. Molecular dating analysis using the concatenated data set was conducted using Least Squares Dating (LSD2) in IQ-TREE 2 with calibration from previously published fossil and codivergence data (split between human and chimpanzee lice 5–7 Mya, split between the lice from Old World primates and Great Apes 20–25 Mya, the minimum age for Menoponidae of 44 Mya based on a fossil; [44,56]) and root age 127.1 Mya [53]. Ancestral states of the mitogenome (single-chromosome or fragmented) were reconstructed over the dated tree using the *ace* function of the APE v5.4 R package [60] under various models: Equal-Rates (ER), All-Rates-Different (ARD), and a model that does not allow transition from fragmented to single-chromosome mitogenome organisation (USR). The best model was selected using the corrected Akaike Information Criterion (AICc) and Akaike Information Criterion weight. The model fit was assessed with the *fitDiscrete* function in the geiger v2.0.7 R package [61], and the best model (ER; AIC = 109.8825, AICc = 109.928, AIC weight = 0.4197; S18 Table) revealed an almost 50% relative likelihood of fragmented ancestral amblyceran mitogenome. Given that more distant outgroups among Psocodea have non-fragmented mitogenomes, a fragmented ancestral state seems unlikely. Therefore, we also performed stochastic mapping with 1000 simulations for the USR models using the phytools v0.7 R package [62]. In addition to maximum likelihood character reconstruction, we also used parsimony to reconstruct the ancestral states (fragmented or non-fragmented) across the tree using the software Mesquite v3.81 [63].

To measure the strength of the phylogenetic signal of fragmentation, we calculated the D-statistic using the *comparative*.*data* and *phylo*.*d* functions of the caper v1.0.1 R package [64] over the dated amblyceran tree.

### Rate of fragmentation

To measure the rate of fragmentation and whether this rate changed over the tree, we analysed the dated concatenated tree in BAMM using Markov chain Monte Carlo (MCMC) simulations [65]. Although this program has become a subject of critique [66], this controversy applies only to quantifying speciation and diversification, not to analyses of phenotypic trait evolution. To set priors for BAMM MCMC cycles and to visualise BAMM outputs, we used the BAMMtools R package [67]. We ran the entire BAMM analysis in three iterations, with the following priors as calculated by BAMMtools: expectedNumberOfShifts = 1.0, betaInitPrior = 0.0136706518546954, betaShiftPrior = 2.90363806597022, useObservedMinMaxAsTraitPriors = 1. The MCMC simulations were run for 10 million generations each, with sampling every one thousand generations. We ran four Markov chains, proposed a chain swap every 1,000 generations, and reset the acceptance rate calculation every 1,000 generations.

### Comparison of branch lengths

The many independent transitions between single-chromosome versus fragmented mitogenome structure in Amblycera (see Results) provided an excellent opportunity to test for the correlation of other factors with mitogenome fragmentation. One main expectation is that fragmented lineages evolve more rapidly [32], which should lead to longer reconstructed branch lengths in fragmented versus non-fragmented taxa. We compared branch lengths, both nuclear and mitochondrial, of taxa with single-chromosome versus fragmented mitogenomes to test whether fragmented mitogenomes evolve faster than the single-chromosome ones. To obtain the nuclear branch lengths, we used the concatenated tree from the phylogenomic analysis, which provides branch lengths in units of substitutions per site for all protein-coding positions under the GTR+G likelihood model. To obtain mitochondrial branch lengths, the amino-acid sequences of mitochondrial protein-coding genes were aligned using UPP [68] with the -M -1 argument to filter out fragmentary sequences. Individual gene alignments were trimmed in trimAl [69] with a gap threshold of 0.4, back-translated using PAL2NAL [70], and concatenated using AMAS [71]. With the concatenated mitochondrial alignment, we estimated mitochondrial branch lengths by constraining the well supported tree from the nuclear gene set (see Results) and using maximum likelihood based on the optimal model (-m MFP) to estimate substitutions per site across all codon positions in the protein-coding genes in IQ-TREE 2 v2.1.2 [57].

To compare branch lengths, we used the Independent Matched Pair Comparison technique [72,73]. Specifically, we used a combination of sister pair comparisons and matched non-overlapping independent paired comparisons to construct contrasts between fragmented and non-fragmented lineages. In cases where a taxon was sister to a clade containing more than one taxon with the relevant mitogenome architecture (as in analogous situations in [73]), we selected the taxon with the median branch length for that clade. In these cases, we scored whether the branch length to the most recent common ancestor for each pair was longer for the fragmented or non-fragmented lineage and compared these fractions using a two-tailed sign test, which is conservative in that it considers only the direction of difference rather than the magnitude [74].

### Ancestral gene orders and gene rearrangements

Given the many independent comparisons available for fragmented and non-fragmented taxa, we were also able to test for a correlation between mitogenome structure and gene rearrangement. As with comparisons of branch lengths (above), we used Independent Matched Pair Comparisons [72,73] to select independent comparisons between fragmented and non-fragmented lineages. These comparisons were constructed to compare whether the number of gene rearrangements, compared to a reconstructed common ancestor, was higher in fragmented versus non-fragmented lineages. In cases where a selection of more than one representative of a clade was possible, we selected the taxon with the number of annotated mitochondrial genes nearest to that of the other taxon to which the comparison was being made. Across the concatenated tree, we reconstructed ancestral mitochondrial gene orders using AGORA v3.1 [75]. In each pair, we compared the numbers of shared gene boundaries shared with the most recent common ancestor. The shared gene boundaries were identified manually, as described by Feng et al. [32]. Because the numbers of annotated mitochondrial genes often differed in each pair member, we divided the number of shared gene boundaries by the number of annotated genes. We then tested whether a higher fraction of lineages with fragmented mitogenomes also had more gene rearrangements than non-fragmented lineages using a two-tailed sign test [74].

### Nucleotide composition

We calculated the nucleotide composition of the mitogenome for six sub-datasets for each sample (all sites, coding regions, different codon positions for all three positions, and fourfold degenerate sites of concatenated PCGs) using the *Bio* and *Bio*.*SeqUtils* packages of Biopython 1.80 [76]. We identified the fourfold degenerate sites using MEGA11 [77], which assigns third position sites as being part of a fourfold or twofold degenerate codon. We performed statistical comparisons of AT content using the ggpubr v0.40 R package [78], the *phylANOVA* function in the phytools v0.7 R package [62], taking into account the concatenated amblyceran tree. Additionally, we calculated AT content for each PCG separately. We used the average AT percentage of different PCGs to test for differences between genes with the Wilcoxon rank sum tests in the rstatix v0.7.2 R package [78]. We also assessed the correlation between AT content and the length of mitochondrial chromosomes. One analysis included the length of all chromosomes regardless of whether they were from single-chromosome of fragmented mitogenomes. The second included only chromosomes from fragmented mitogenomes. For these regressions, we fit both linear and Phylogenetic Least Squares (PGLS) models to the total AT content and AT content of fourfold degenerate sites, taking into account the concatenated amblyceran tree. For fragmented mitogenomes, we tested both the average length of chromosomal fragments for each species, and also using the length of all chromosomes as separate data points. For PGLS, we employed the *pgls* function of the CAPER v1.0.1 R package with both Brownian and Pagel’s λ correlations.

## Supporting information

S1 Fig Concatenated maximum likelihood phylogenomic tree of Amblycera. Based on a target set of 2395 protein-coding genes. Numbers associated with branches indicate ultrafast bootstrap support.
(TIF)

S2 Fig Coalescent tree of Amblycera. Based on ASTRAL analysis of a target set of 2395 protein-coding genes, combining individual gene trees into a species tree. Numbers associated with branches indicate local posterior probability.
(TIF)

S3 Fig Dated phylogenomic tree of Amblycera. Based on concatenated data set and a target set of 2395 protein-coding genes. Numbers at branches indicate 95% confidence intervals.
(TIF)

S4 Fig Dated phylogenomic tree with ancestral state reconstruction of mitogenome evolution in Amblycera under the Equal Rates (ER) model. Pie charts at the nodes show the frequency distribution of reconstructed ancestral state after 1000 simulations of stochastic character mapping using an equal rates (ER) model. The ER model was best fitting according to the Akaike Information Criterion and Akaike Information Criterion weight (AIC = 109.8825, AIC weight = 0.4197). Circles at the tips indicate mitogenome structure (single-chromosome versus fragmented). Time scale at bottom in million years ago (Mya).
(TIF)

S5 Fig Phylogenetic signal of the mitogenome state of Amblycera (fragmented versus single-chromosome). Blue is values of D under simulation using Browning motion. Red is values of D under simulation with random phylogenetic structure. Black vertical line indicates value of D over actual tree (D = 0.281), *P* of E(D) under Brownian motion model = 0.177, *P* of E(D) under no phylogenetic structure model = 0.001.
(TIF)

S6 Fig Ancestral mitogenome states of Amblycera reconstructed with parsimony and concatenated maximum likelihood phylogenomic tree. White circles indicate single-chromosome mitogenomes, black circles indicate fragmented mitogenomes.
(TIF)

S7 Fig Bayesian credible set of configurations showing changes in fragmentation rate in mitogenomes of Amblycera. Circles at the branches indicate rate shift events included in nine most probable reconstructions, numbers above the plots indicate posterior probabilities. Overall reconstructed rate on each branch shown with color from low rate (dark blue) to high rate (red) as indicated by the scale bar in Fig 4.
(TIF)

S8 Fig Pairs of Amblycera selected for comparison of gene rearrangements. Circles at the tips indicate mitogenome structure (single-chromosome versus fragmented), and samples compared within a pair connected with blue line. Codes at the nodes indicate ancestral genomes, as in S20 and S21 Tables. Both topology and branch lengths preserved from the nuclear concatenated tree.
(TIF)

S9 Fig Comparison of AT content of fragmented (red) and single-chromosome (green) mitogenomes of Amblycera. Values calculated from both coding and non-coding regions. Centerline–median; box limit–upper and lower quartiles; whiskers—interquartile range. The black dotted line shows the average percent AT content for insect mitogenomes available on NCBI GenBank (76.0%).
(TIF)

S10 Fig Percent AT content of different partitions of amblyceran mitogenomes. Categories indicates values for entire sequences, coding regions, different codon positions, and fourfold degenerate sites. Centerline–median; box limit–upper and lower quartiles; whiskers—interquartile range. Significance based on Wilcoxon rank sum tests.
(TIF)

S11 Fig Percent AT content of different partitions of single-chromosome and fragmented amblyceran mitogenomes. Categories indicate values for entire sequences, coding regions, different codon positions, and fourfold degenerate sites. Centerline–median; box limit–upper and lower quartiles; whiskers—interquartile range. The black dotted line shows the average percent AT content for insect mitogenomes available on NCBI GenBank (76.0%).
(TIF)

S12 Fig Percent AT content of different amblyceran protein-coding genes. Showing differences between entire sequences and fourfold degenerate sites. Centerline–median; box limit–upper and lower quartiles; whiskers—interquartile range. The black dotted line indicates the average AT content for insect mitogenomes (entire sequences) available in NCBI GenBank (76.0%).
(TIF)

S1 Table Summary of analysed mitogenomes of Amblycera, including accession numbers for the original raw data used for assembly. Previously published raw reads are in grey; all samples were newly assembled. NC—number of chromosomes.
(XLSX)

S2 Table Gene order of analysed mitogenomes. *at the beginning of a fragment–this fragment was not considered circular. +at the end of a fragment–more genes were cut away during the circularization process by AWA. NC—number of chromosomes.
(XLSX)

S3 Table Subsampling used for assembly of amblyceran mitogenomes and methods used for chromosome circularization. Each fragment has a separate line, grey fragments were not considered circular.
(XLSX)

S4 Table Mononucleotide repeats of 18 bp or longer found in mitogenome assemblies of Amblycera. Numbers in columns indicate length of respective chromosome and numbers of A and T repeats, respectively.
(XLSX)

S5 Table AT contents of atp6 genes. Computed from entire gene sequences, different codon sets, and fourfold degenerate sites.
(XLSX)

S6 Table AT contents of atp8 genes. Computed from entire gene sequences, different codon sets, and fourfold degenerate sites.
(XLSX)

S7 Table AT contents of cob genes. Computed from entire gene sequences, different codon sets, and fourfold degenerate sites.
(XLSX)

S8 Table AT contents of cox1 genes. Computed from entire gene sequences, different codon sets, and fourfold degenerate sites.
(XLSX)

S9 Table AT contents of cox2 genes. Computed from entire gene sequences, different codon sets, and fourfold degenerate sites.
(XLSX)

S10 Table AT contents of cox3 genes. Computed from entire gene sequences, different codon sets, and fourfold degenerate sites.
(XLSX)

S11 Table AT contents of nad1 genes. Computed from entire gene sequences, different codon sets, and fourfold degenerate sites.
(XLSX)

S12 Table AT contents of nad2 genes. Computed from entire gene sequences, different codon sets, and fourfold degenerate sites.
(XLSX)

S13 Table AT contents of nad3 genes. Computed from entire gene sequences, different codon sets, and fourfold degenerate sites.
(XLSX)

S14 Table AT contents of nad4 genes. Computed from entire gene sequences, different codon sets, and fourfold degenerate sites.
(XLSX)

S15 Table AT contents of nad4l genes. Computed from entire gene sequences, different codon sets, and fourfold degenerate sites.
(XLSX)

S16 Table AT contents of nad5 genes. Computed from entire gene sequences, different codon sets, and fourfold degenerate sites.
(XLSX)

S17 Table AT contents of nad6 genes. Computed from entire gene sequences, different codon sets, and fourfold degenerate sites.
(XLSX)

S18 Table Aikake Information Criteria (AIC), corrected Aikake Information Criteria (AICc) and AIC weights for ancestral mitogenome state reconstruction models. ER—equal rates model, ARD–all-rates-different model, USR—irreversible fragmentation model.
(XLSX)

S19 Table Comparison of mitochondrial and nuclear branch lengths in single-chromosome and fragmented mitogenomes. Computed from topography of nuclear concatenated tree.
(XLSX)

S20 Table Comparison of gene rearrangements in single-chromosome and fragmented mitogenomes. Rearrangements counted from most recent common ancestor and divided by numbers of annotated genes. MRCA—most recent common ancestor. Position of ancestral nodes shown in S8 Fig.
(XLSX)

S21 Table Ancestral gene orders of Amblycera inferred from concatenated tree. Position of ancestral nodes shown in S8 Fig.
(XLSX)

S22 Table AT contents in analysed mitogenomes. Computed from entire sequences, coding regions, different codon sets, and fourfold degenerate sites.
(XLSX)

S23 Table Statistics of differences between AT contents of separate protein-coding genes. P-values and significance are the results of Wilcoxon rank sum tests.
(XLSX)

S24 Table Completeness of individual nuclear gene alignments. Single letters in headings of columns indicate numbers of respective bases: A–Adenine; C–Cytosine; G–Guanine; T–Thymine; N–base present but not determined;—–base missing.
(XLSX)
