# Supplementary figures and images for: Mitochondrial genome fragmentation is correlated with increased rates of molecular evolution
Source: PLoS Genet. 2024 May 3;20(5):e1011266. doi: 10.1371/journal.pgen.1011266 (PMC11095710; doi:10.1371/journal.pgen.1011266)

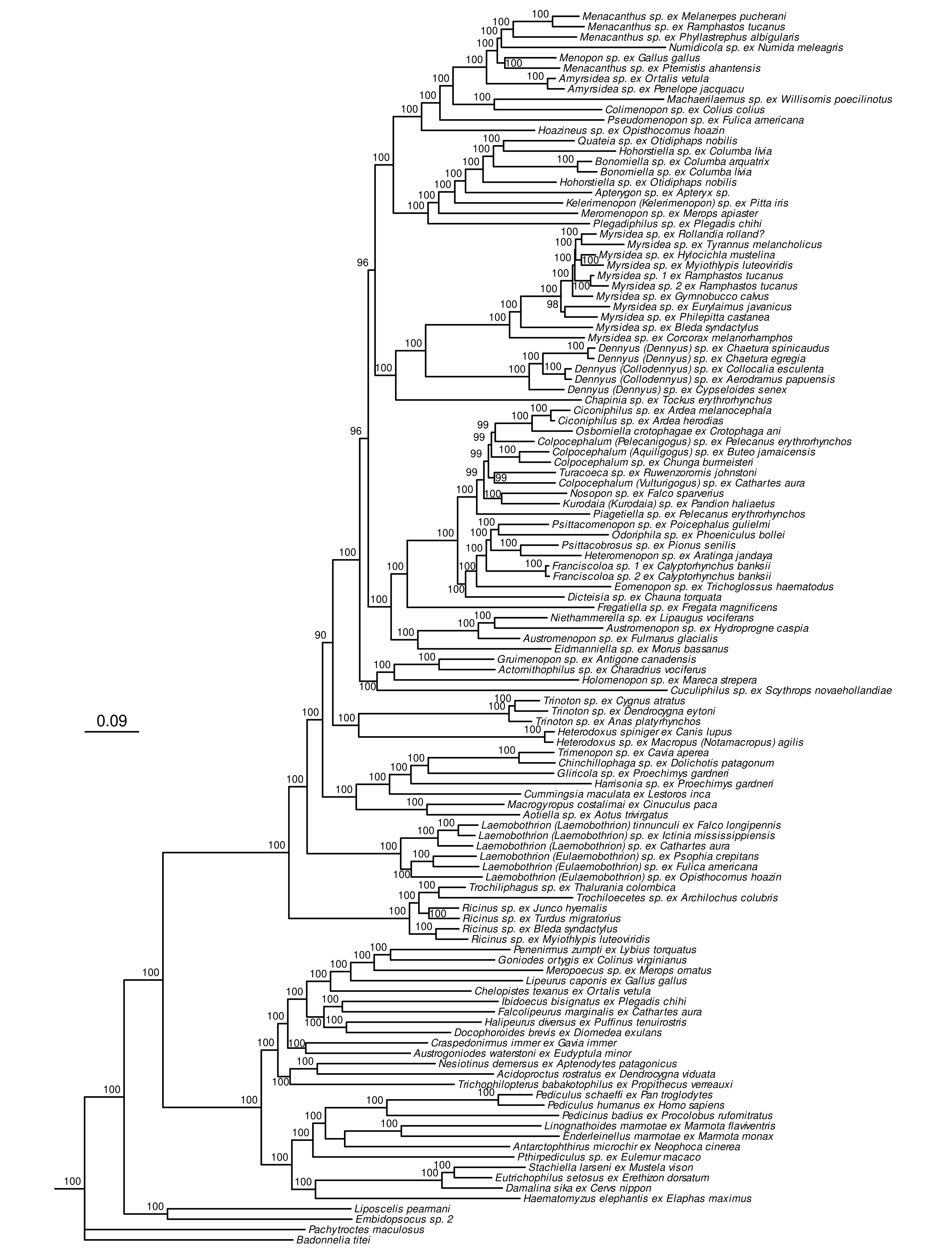

Supplement: S1 Fig — Based on a target set of 2395 protein-coding genes. Numbers associated with branches indicate ultrafast bootstrap support. (TIF) [file pgen.1011266.s001.tif]

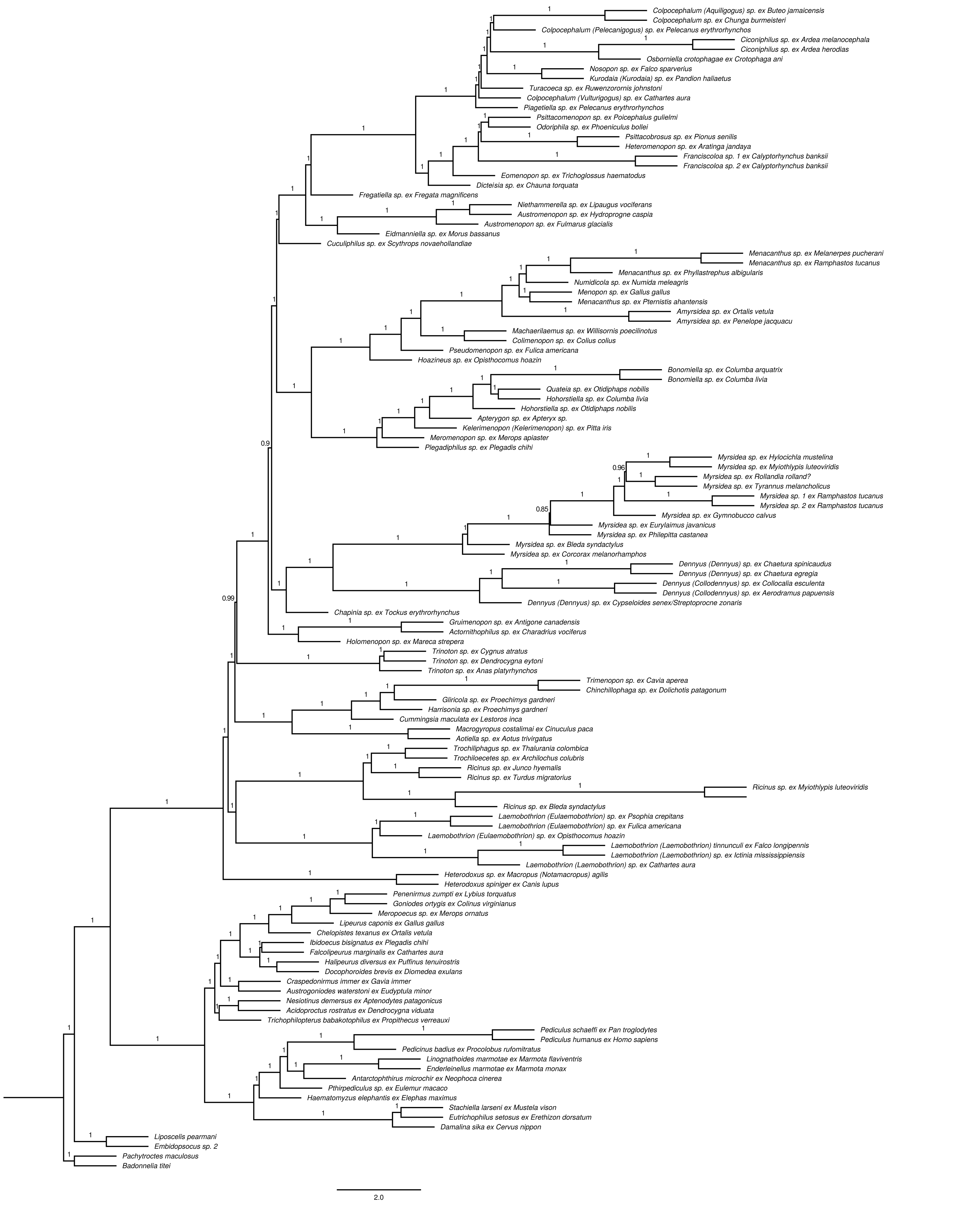

Supplement: S2 Fig — Based on ASTRAL analysis of a target set of 2395 protein-coding genes, combining individual gene trees into a species tree. Numbers associated with branches indicate local posterior probability. (TIF) [file pgen.1011266.s002.tif]

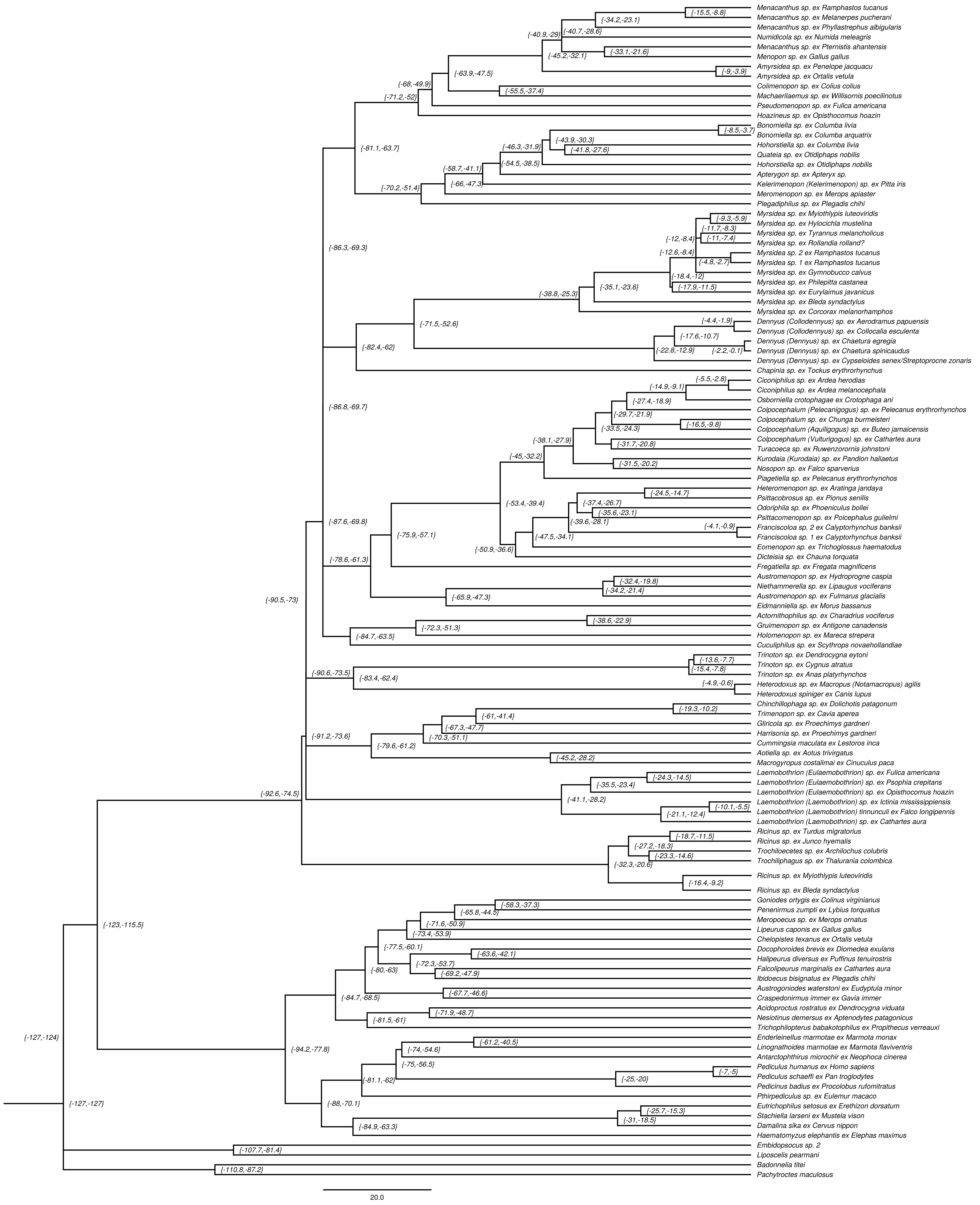

Supplement: S3 Fig — Based on concatenated data set and a target set of 2395 protein-coding genes. Numbers at branches indicate 95% confidence intervals. (TIF) [file pgen.1011266.s003.tif]

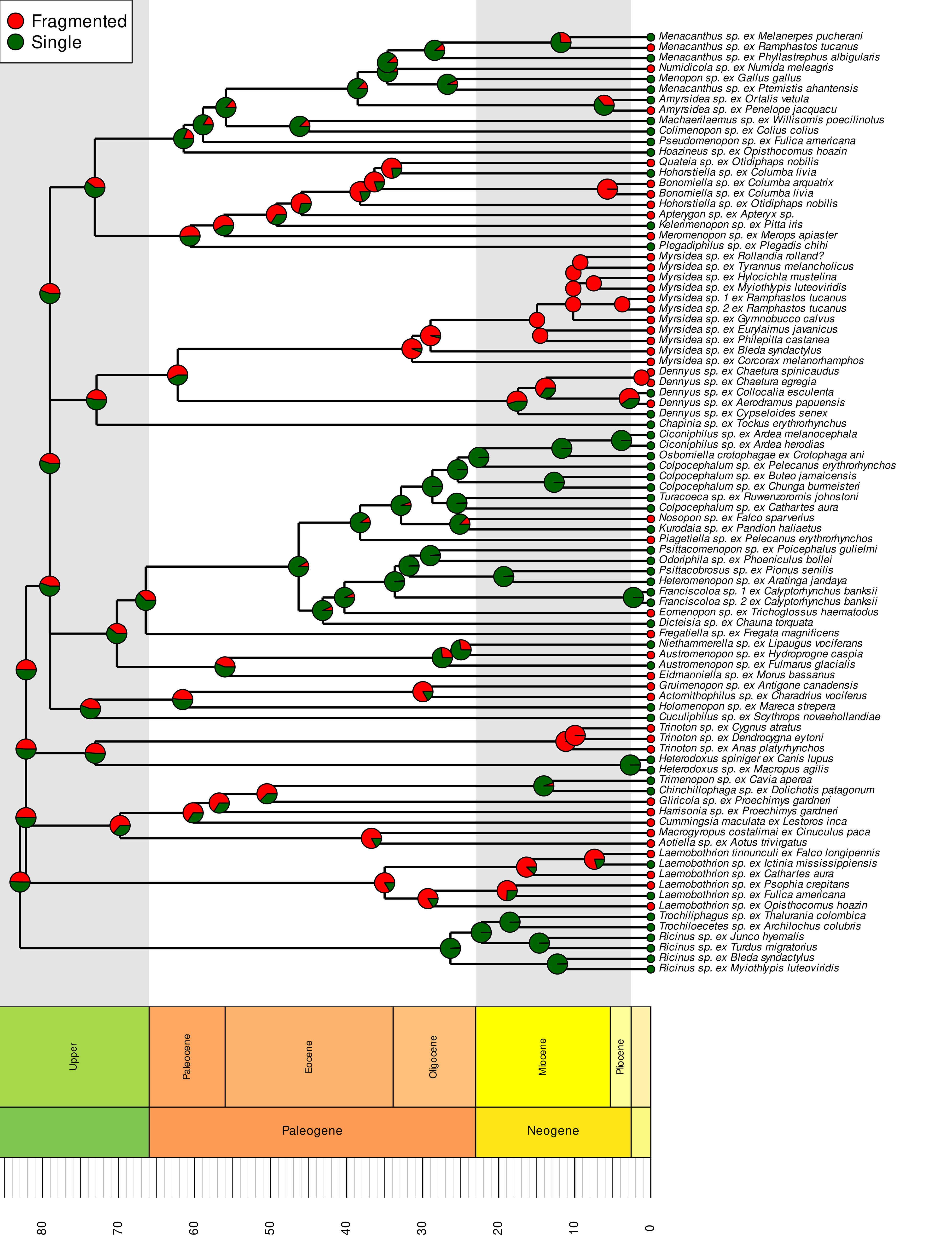

Supplement: S4 Fig — Pie charts at the nodes show the frequency distribution of reconstructed ancestral state after 1000 simulations of stochastic character mapping using an equal rates (ER) model. The ER model was best fitting according to the Akaike Information Criterion and Akaike Information Criterion weight (AIC = 109.8825, AIC weight = 0.4197). Circles at the tips indicate mitogenome structure (single-chromosome versus fragmented). Time scale at bottom in million years ago (Mya). (TIF) [file pgen.1011266.s004.tif]

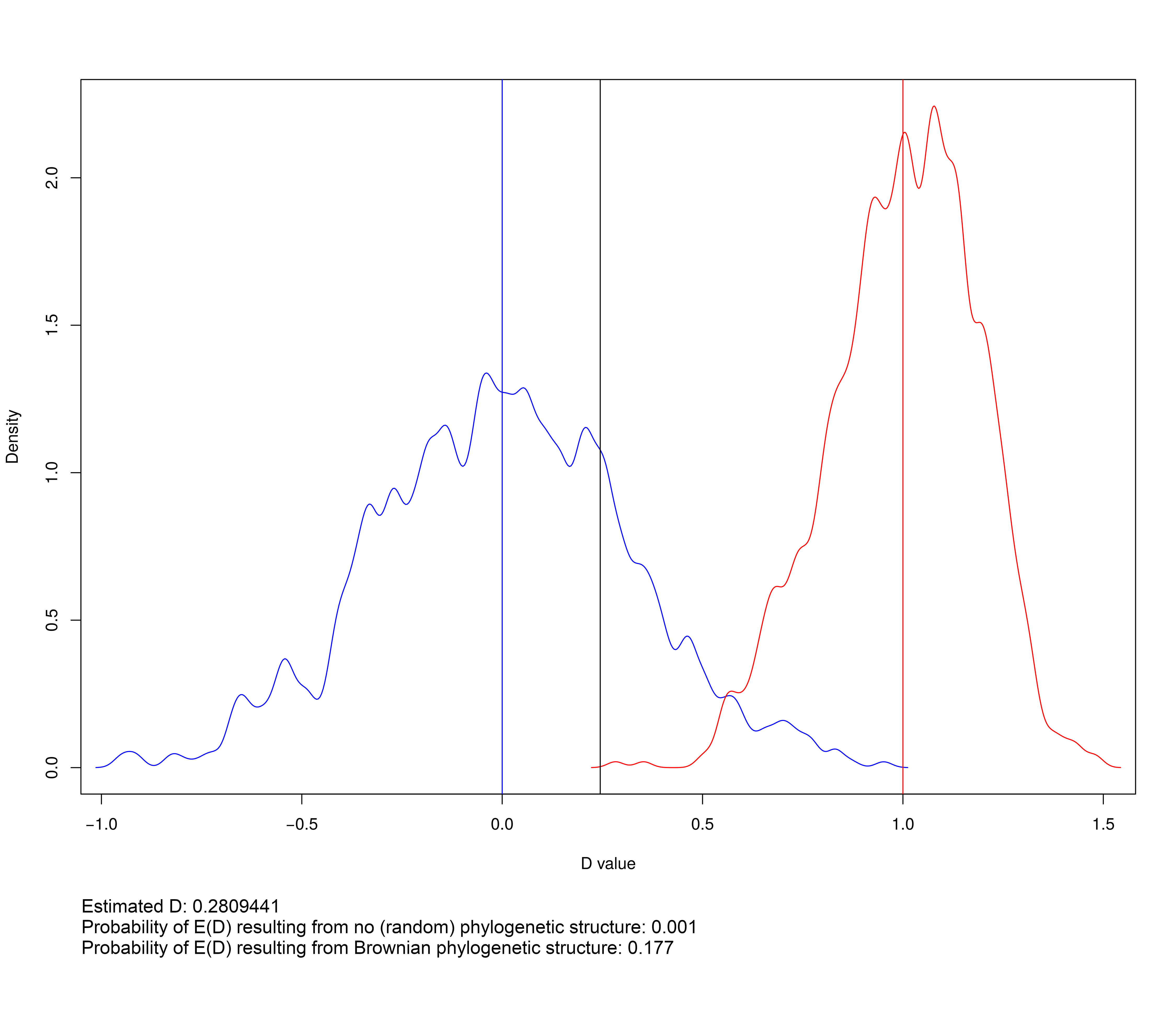

Supplement: S5 Fig — Blue is values of D under simulation using Browning motion. Red is values of D under simulation with random phylogenetic structure. Black vertical line indicates value of D over actual tree (D = 0.281), P of E(D) under Brownian motion model = 0.177, P of E(D) under no phylogenetic structure model = 0.001. (TIF) [file pgen.1011266.s005.tif]

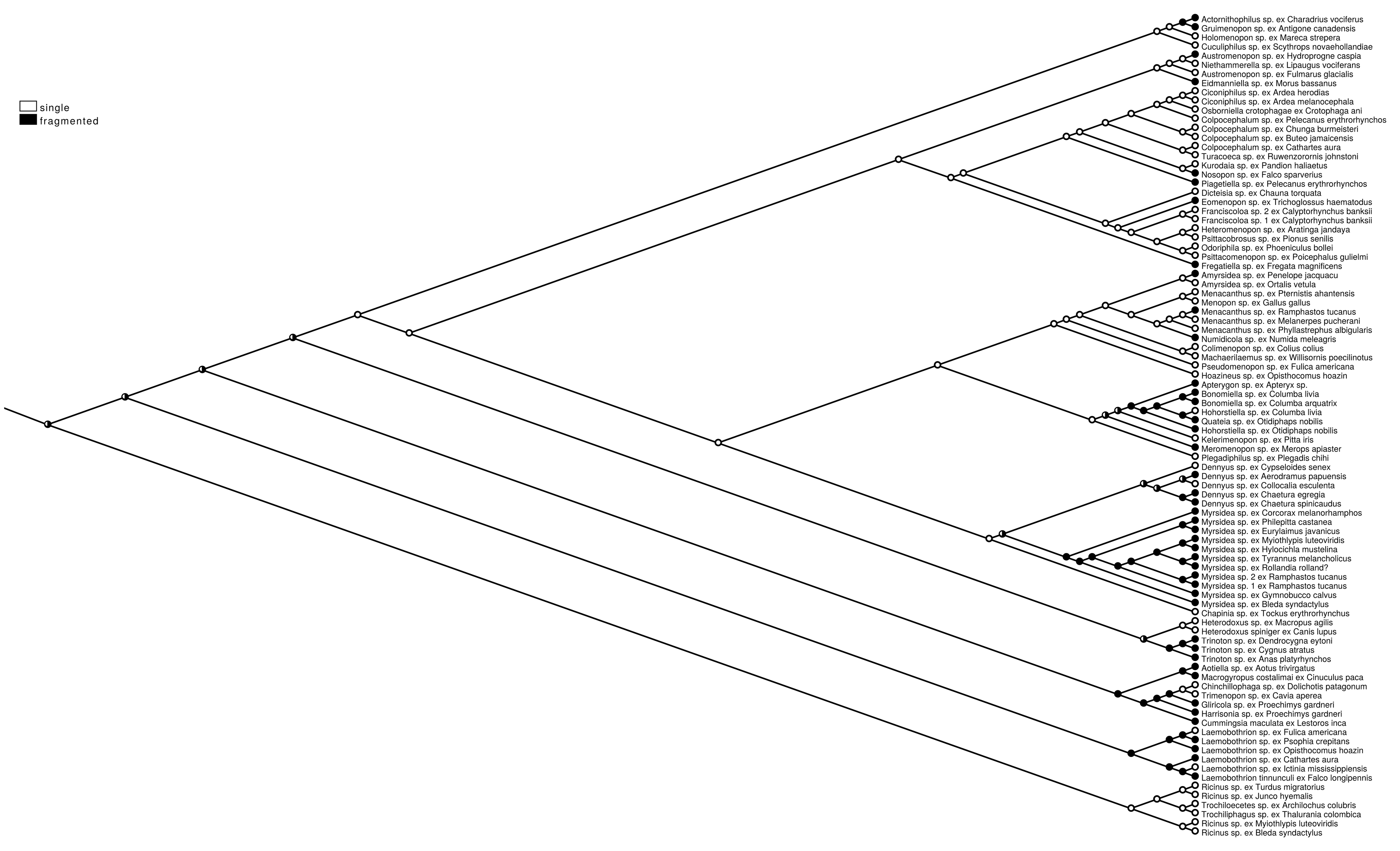

Supplement: S6 Fig — White circles indicate single-chromosome mitogenomes, black circles indicate fragmented mitogenomes. (TIF) [file pgen.1011266.s006.tif]

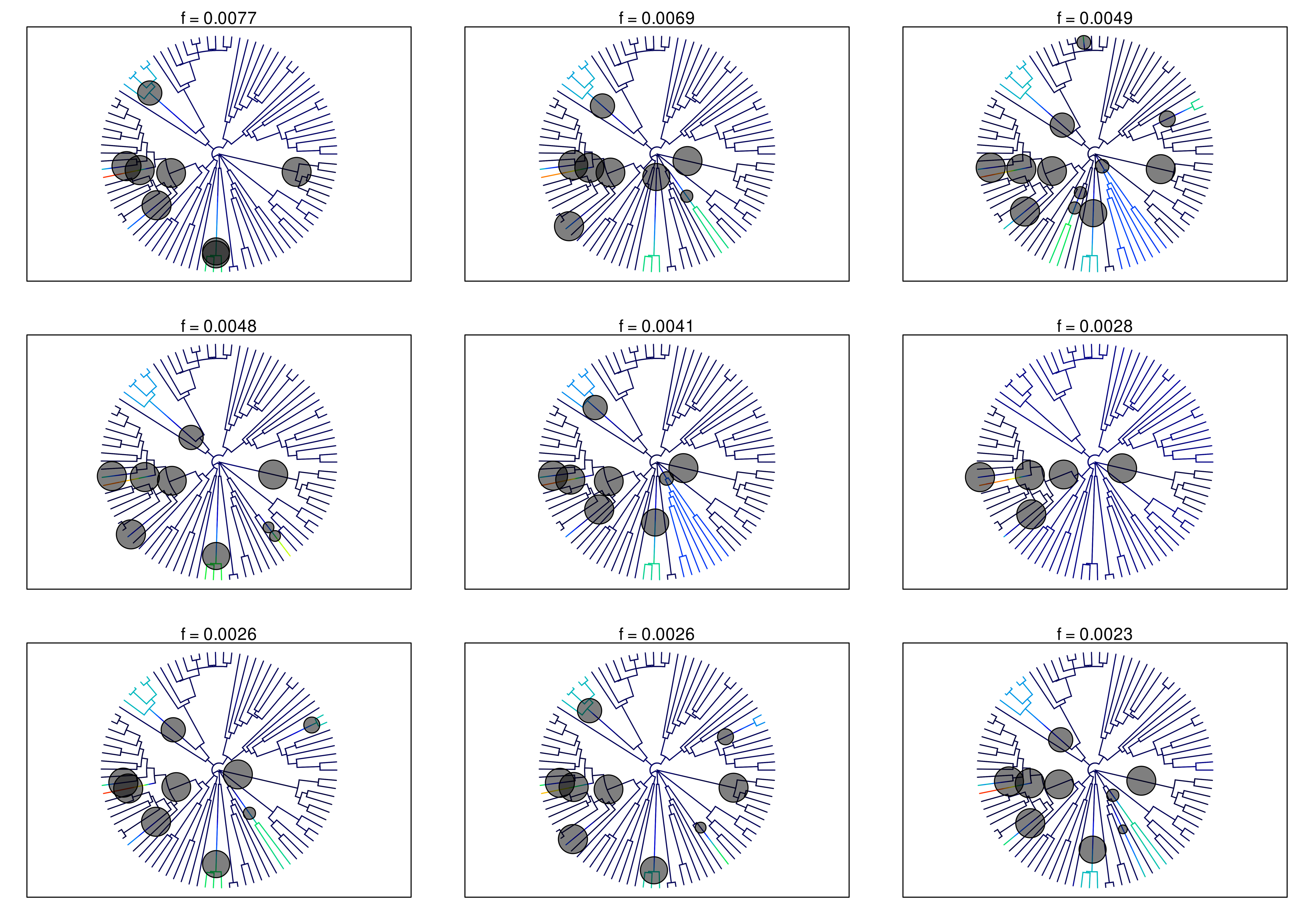

Supplement: S7 Fig — Circles at the branches indicate rate shift events included in nine most probable reconstructions, numbers above the plots indicate posterior probabilities. Overall reconstructed rate on each branch shown with color from low rate (dark blue) to high rate (red) as indicated by the scale bar in Fig 4. (TIF) [file pgen.1011266.s007.tif]

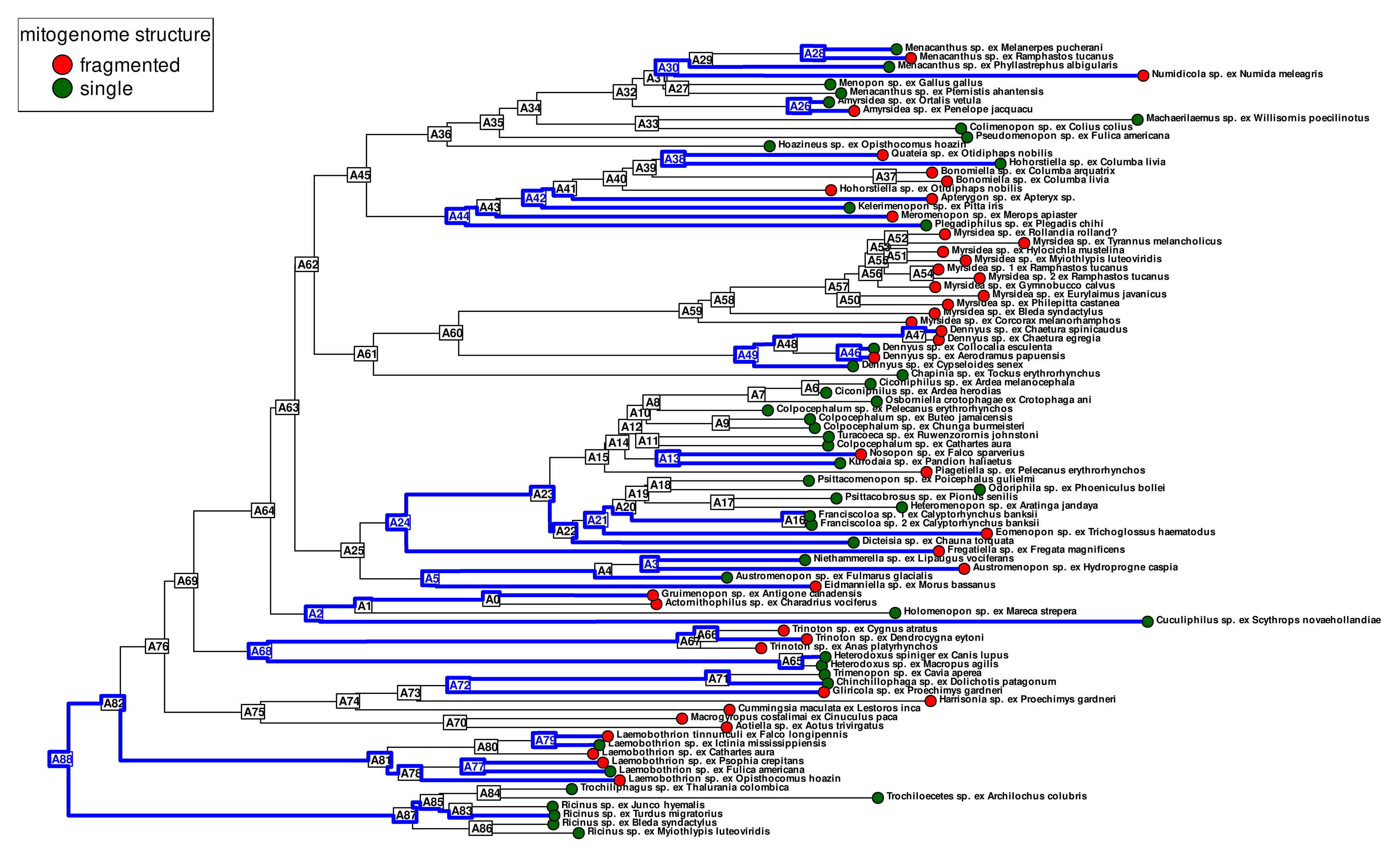

Supplement: S8 Fig — Circles at the tips indicate mitogenome structure (single-chromosome versus fragmented), and samples compared within a pair connected with blue line. Codes at the nodes indicate ancestral genomes, as in S20 and S21 Tables. Both topology and branch lengths preserved from the nuclear concatenated tree. (TIF) [file pgen.1011266.s008.tif]

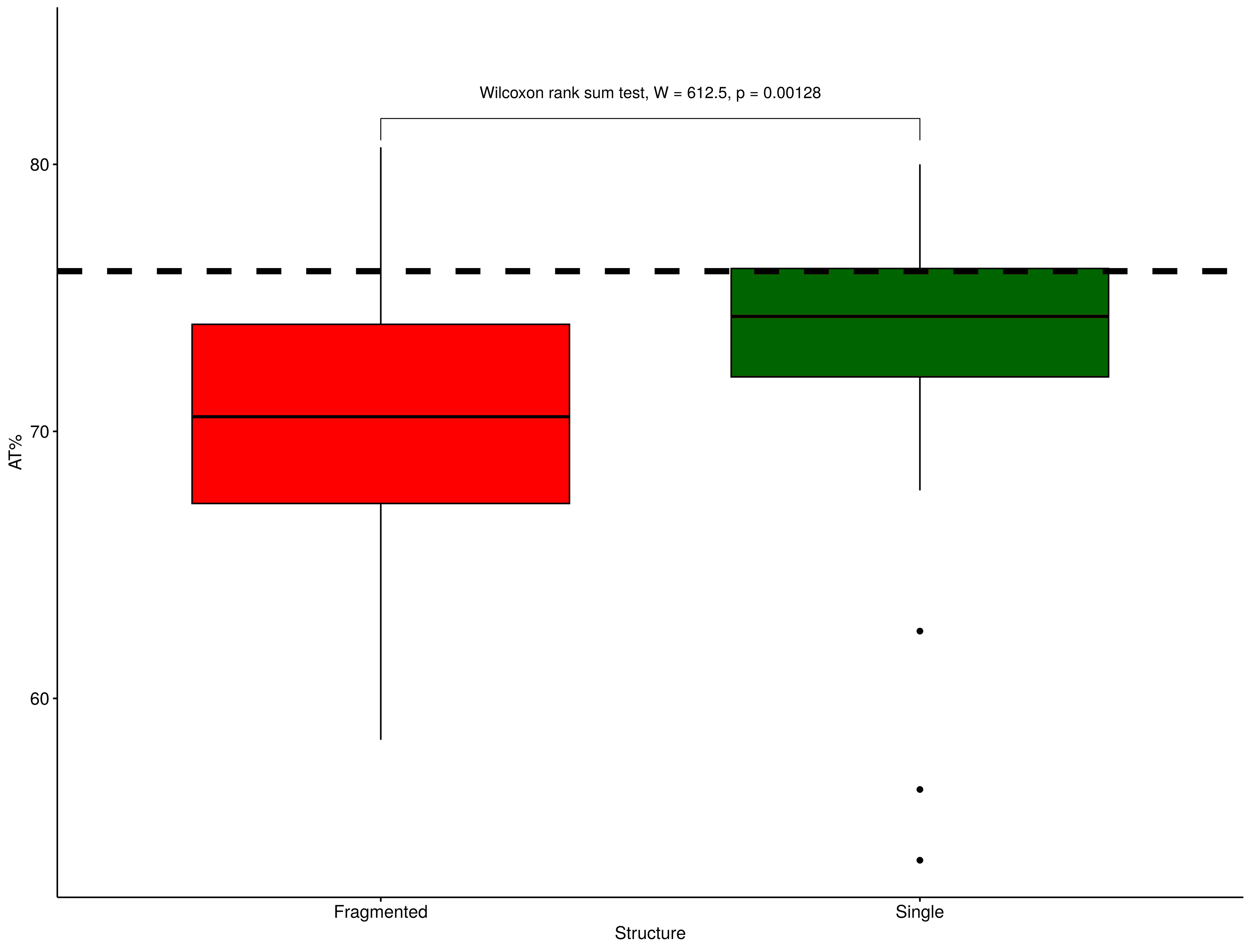

Supplement: S9 Fig — Values calculated from both coding and non-coding regions. Centerline–median; box limit–upper and lower quartiles; whiskers—interquartile range. The black dotted line shows the average percent AT content for insect mitogenomes available on NCBI GenBank (76.0%). (TIF) [file pgen.1011266.s009.tif]

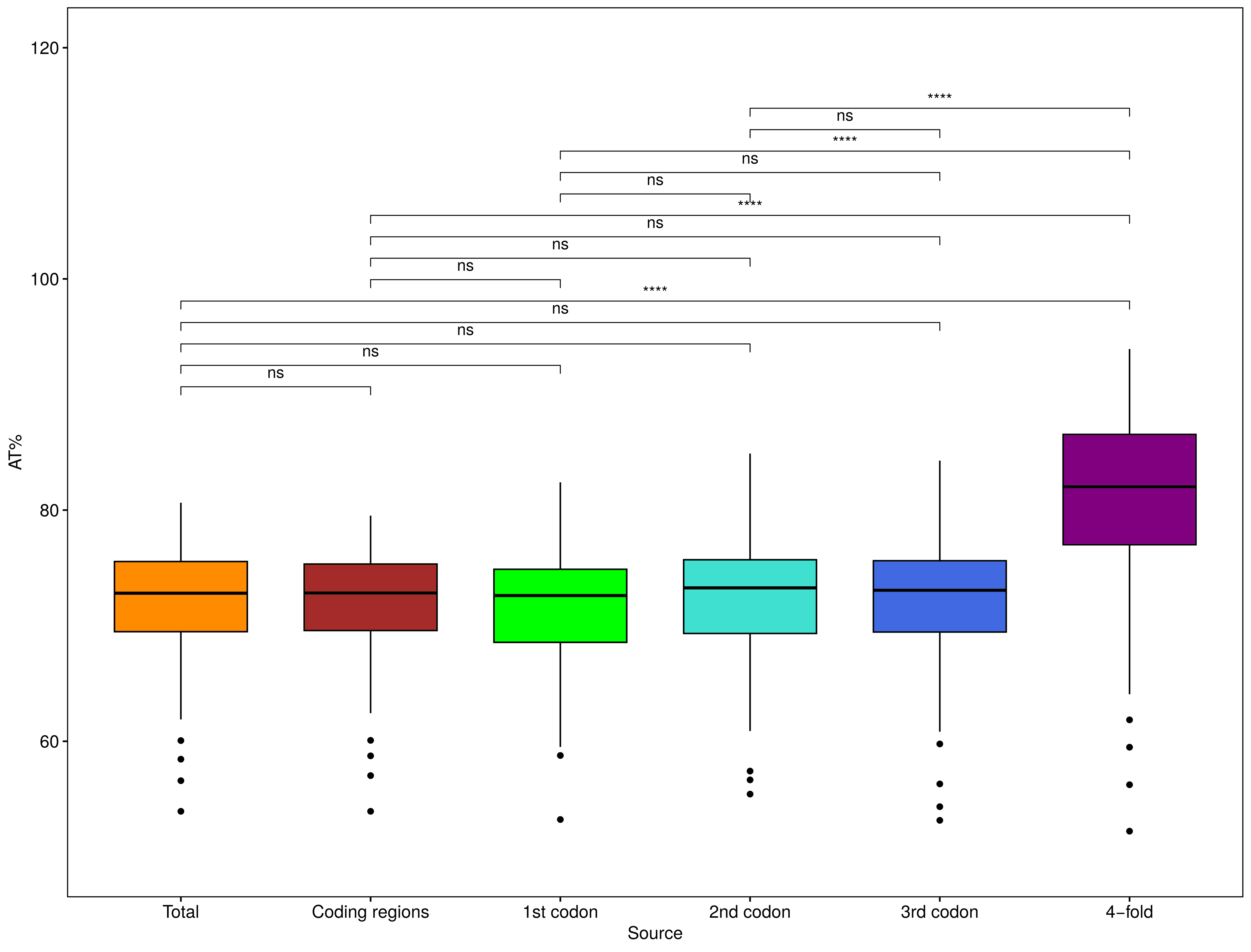

Supplement: S10 Fig — Categories indicates values for entire sequences, coding regions, different codon positions, and fourfold degenerate sites. Centerline–median; box limit–upper and lower quartiles; whiskers—interquartile range. Significance based on Wilcoxon rank sum tests. (TIF) [file pgen.1011266.s010.tif]

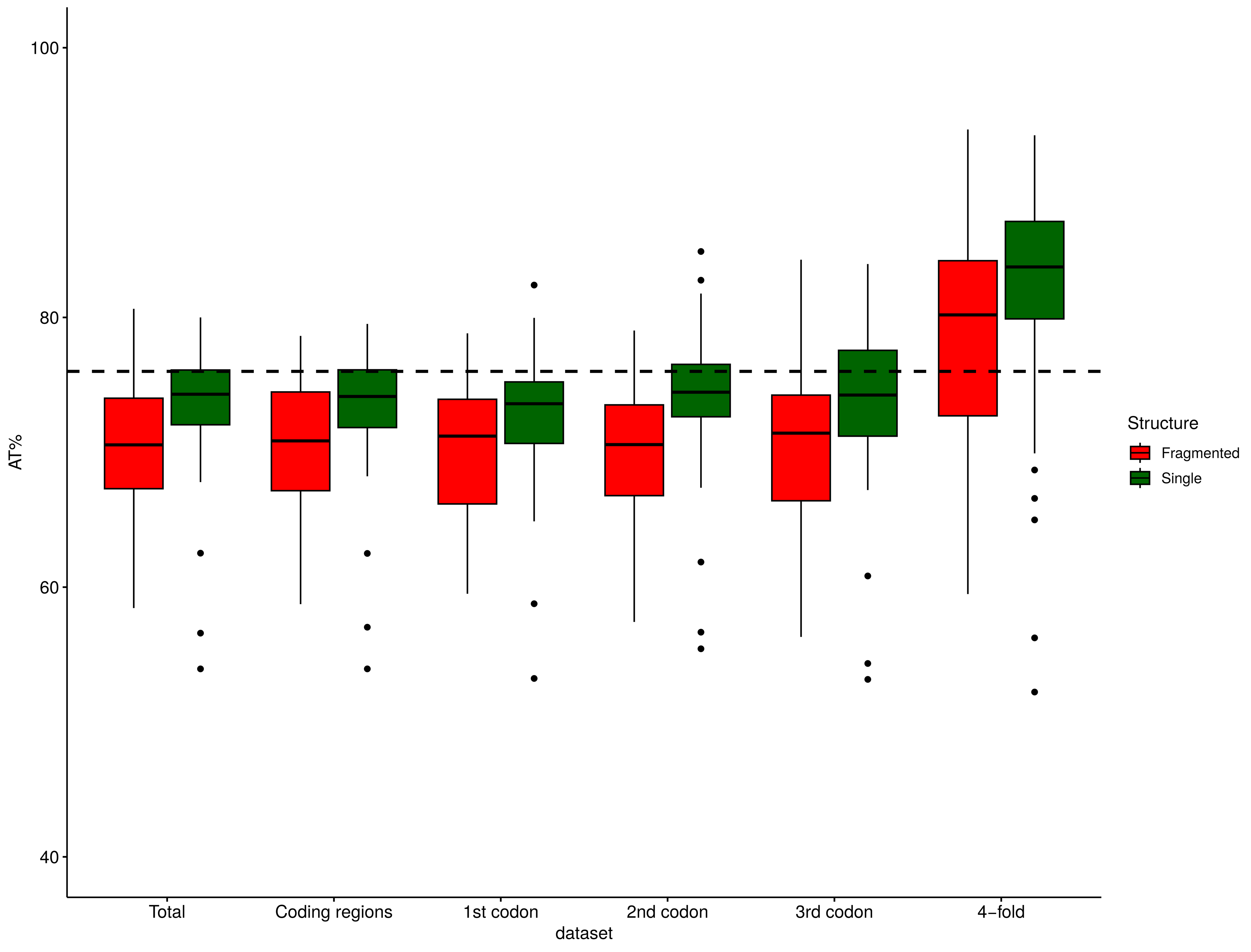

Supplement: S11 Fig — Categories indicate values for entire sequences, coding regions, different codon positions, and fourfold degenerate sites. Centerline–median; box limit–upper and lower quartiles; whiskers—interquartile range. The black dotted line shows the average percent AT content for insect mitogenomes available on NCBI GenBank (76.0%). (TIF) [file pgen.1011266.s011.tif]

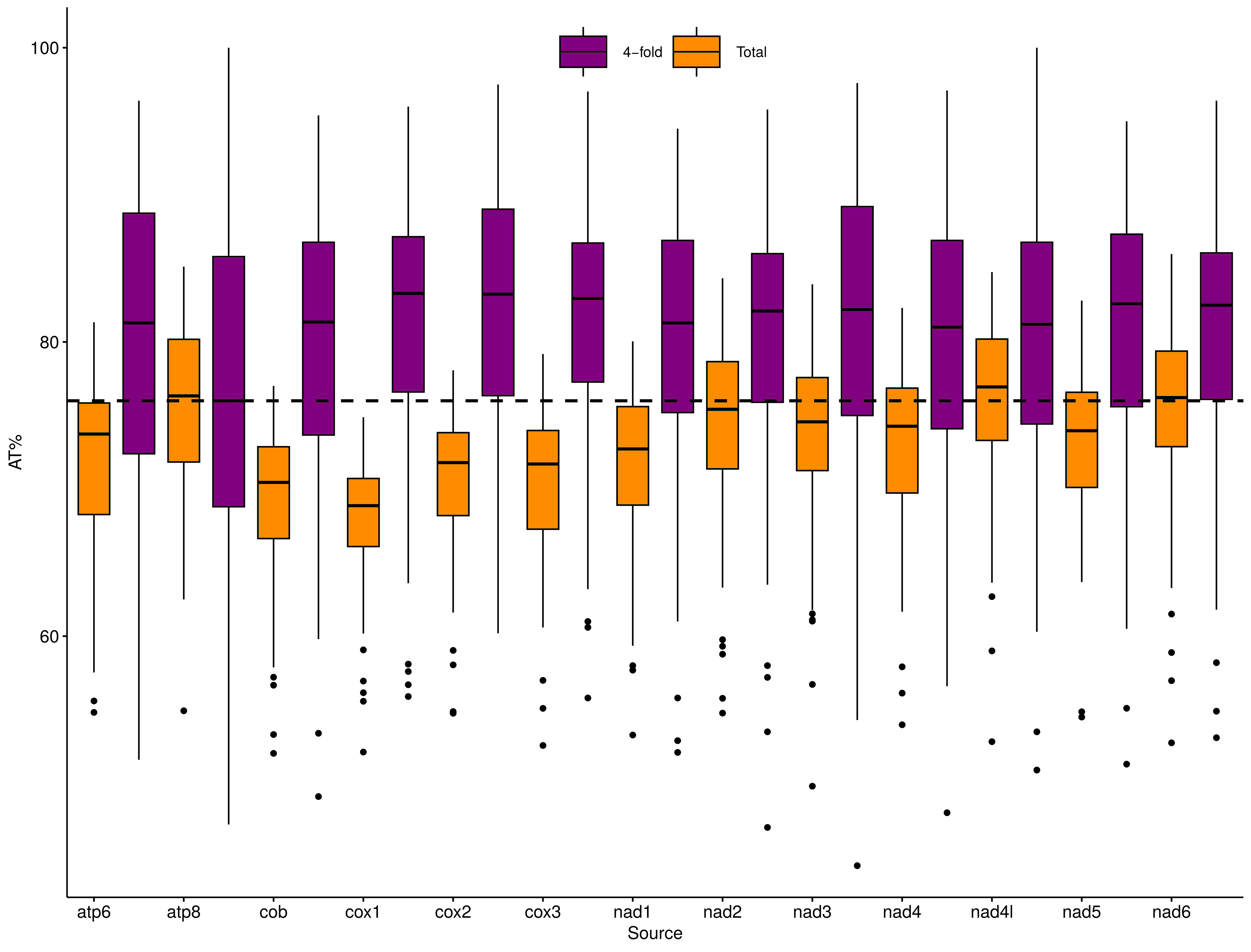

Supplement: S12 Fig — Showing differences between entire sequences and fourfold degenerate sites. Centerline–median; box limit–upper and lower quartiles; whiskers—interquartile range. The black dotted line indicates the average AT content for insect mitogenomes (entire sequences) available in NCBI GenBank (76.0%). (TIF) [file pgen.1011266.s012.tif]
